# Supplementary material for: Daily struggle to take antiretrovirals: a qualitative study in Papuans living with HIV and their healthcare providers
Source: BMJ Open. 2020 Sep 28;10(9):e036832. doi: 10.1136/bmjopen-2020-036832 (PMC7523200; doi:10.1136/bmjopen-2020-036832)
Supplement: Author's manuscript [file bmjopen-2020-036832.draft_revisions.pdf]

BMJ Open is committed to open peer review. As part of this commitment we make the peer review history of every article we publish publicly available.

When an article is published we post the peer reviewers' comments and the authors' responses online. We also post the versions of the paper that were used during peer review. These are the versions that the peer review comments apply to.

The versions of the paper that follow are the versions that were submitted during the peer review process. They are not the versions of record or the final published versions. They should not be cited or distributed as the published version of this manuscript.

BMJ Open is an open access journal and the full, final, typeset and author-corrected version of record of the manuscript is available on our site with no access controls, subscription charges or pay-per-view fees (http://bmjopen.bmj.com).

If you have any questions on BMJ Open's open peer review process please email info.bmjopen@bmj.com

BMJ Open

The daily struggle to take antiretrovirals: a qualitative study in Papuans living with HIV and their healthcare providers

Journal:	*BMJ Open*
Manuscript ID	bmjopen-2020-036832
Article Type:	Original research
Date Submitted by the Author:	13-Jan-2020
Complete List of Authors:	Sianturi, Elfride; Groningen University Faculty of Mathematics and Natural Sciences, Pharmacy; Universitas Cenderawasih,<br>Latifah, E; Universitas Muhammadiyah Magelang<br>Probandari, Ari; Universitas Sebelas Maret, Department of Public Health, Faculty of Medicine<br>Effendy, C; Gadjah Mada University Faculty of Medicine<br>Taxis, Katja; University of Groningen, Faculty of Mathematics and Natural Sciences
Keywords:	HIV & AIDS < INFECTIOUS DISEASES, ETHICS (see Medical Ethics), Quality in health care < HEALTH SERVICES ADMINISTRATION & MANAGEMENT

SCHOLARONE™
Manuscripts

I, the Submitting Author has the right to grant and does grant on behalf of all authors of the Work (as defined in the below author licence), an exclusive licence and/or a non-exclusive licence for contributions from authors who are: i) UK Crown employees; ii) where BMJ has agreed a CC-BY licence shall apply, and/or iii) in accordance with the terms applicable for US Federal Government officers or employees acting as part of their official duties; on a worldwide, perpetual, irrevocable, royalty-free basis to BMJ Publishing Group Ltd ("BMJ") its licensees and where the relevant Journal is co-owned by BMJ to the co-owners of the Journal, to publish the Work in this journal and any other BMJ products and to exploit all rights, as set out in our licence.

The Submitting Author accepts and understands that any supply made under these terms is made by BMJ to the Submitting Author unless you are acting as an employee on behalf of your employer or a postgraduate student of an affiliated institution which is paying any applicable article publishing charge ("APC") for Open Access articles. Where the Submitting Author wishes to make the Work available on an Open Access basis (and intends to pay the relevant APC), the terms of reuse of such Open Access shall be governed by a Creative Commons licence – details of these licences and which Creative Commons licence will apply to this Work are set out in our licence referred to above.

Other than as permitted in any relevant BMJ Author's Self Archiving Policies, I confirm this Work has not been accepted for publication elsewhere, is not being considered for publication elsewhere and does not duplicate material already published. I confirm all authors consent to publication of this Work and authorise the granting of this licence.

For peer review only

The daily struggle to take antiretrovirals: a qualitative study in Papuans living with HIV and their healthcare providers

E.I Sianturi[1,2,] E. Latifah[3], A. Probandari[4], C. Effendy[5], K. Taxis[1]

[1]PharmacoTherapy,-Epidemiology&-Economics (PTEE) Department of Pharmacy,University of Groningen, the Netherlands
[2]Faculty of Mathematics and Natural Sciences University of Cenderawasih, Papua-Indonesia
[3]Department of Pharmacy, Faculty of Health Science, Universitas Muhammadiyah Magelang, Magelang, Indonesia
[4]Department of Public Health, Faculty of Medicine, Universitas Sebelas Maret, Jl. Ir. Sutami 36A, Surakarta, 57126, Indonesia.
[5]School of Nursing, Faculty of Medicine, Public Health and Nursing, Universitas Gadjah Mada, Yogyakarta, Indonesia.

Correspondence should be addressed to:
E.I Sianturi, Antonius Deusinglaan 1, 9713 AV, Groningen, The Netherlands, Contact Phone Number: +31 0622820392, Fax number: +31 503637576; Contact email: e.i.sianturi@rug.nl; ira_sianturi@yahoo.co.id

Email's authors:

E.I Sianturi: ira_sianturi@yahoo.co.id; e.i.sianturi@rug.nl

E. Latifah: elmiawatilatifah@ummgl.ac.id

A. Probandari: ariprobandari@yahoo.com

C. Effendy: christantie@gmail.com

K. Taxis: k.taxis@rug.nl

For peer review only

Abstract

Objective: The study aimed to explore the perspective and the experiences of Papuans, are Melanesians with Christianity as the dominant religion, living with HIV to take ART.

Design: This was a qualitative study covering all five tribes located in Papua Provinces one of two Indonesian provinces on Papua Island. Semi structured interviews were conducted with Papuans living with HIV and their health care providers (HCPs). Interviews were transcribed verbatim and coded to find themes.

Results: Overall, we conducted interviews with 13 Papuans living with HIV (mean age: 33 years, 61 % female) and 14 HCPs (mean age: 42 years, 64% female) within five customary areas. HCPs included three physicians, nine nurses, two others. We found four themes: (1) organization of care, (2) healthcare providers, (3) social support, and (4) patient's knowledge and beliefs. Important factors in those themes were stigma from family, community, and HCPs as well as practical problems such as transportation because of long distance. The HIV treatment strategies implemented in Papua need to be corrected with some attention to social culture of Papuan.

Conclusion: Despite free access to ART, Papuans living with HIV struggle to remain on treatment. Considering local culture and religion, broader strategies to reduce stigma and HIV treatment strategies should be a priority for the centralized and local government after implementation of HIV strategies in Papua has been replicating from the rest of Indonesia area with insufficient interest to social cultural of Papuans.

Keyword: HIV, Papuan, Melanesian, experiences, tribes, customary-area, local culture, stigma, Indonesia

For peer review only

Strengths and Limitations of this study

- Our findings provide new insights into the daily struggle in taking ART among Melanesians in Indonesia.
- We specifically recruited patients from five different tribes in Papua. Other racial/ethnic groups and may have different experiences.
- We asked HCPs to ask patients to participate in this study, so our results refer to patients who remain in chronic care.
- More work needs to be done to identify factors driving patients to drop out of care completely.

For peer review only

Background

Antiretroviral therapy (ART) has saved millions of people living with HIV (PLHIV) [1]. Treatment is lifelong. Despite such successes, access to treatment and retention in care is still an issue in many parts of the world [2]. Economic barriers, stigma, social relationships, religion, local concepts about illness and medication impact on the care for PLHIV [3], [4].

Indonesia consists of more than 17 thousand islands and has been viewed as one of Southeast Asia's highest performing economies in recent decades [5]. A large number of islands has fostered the development of a diverse culture with more than four hundred ethnic groups, each with their own language(s) [5], [6]. The prevalence of HIV is about 0.1% across Indonesia. The prevalence in Papua is about 2.3%, which with the highest prevalence found in the remote areas of the highlands [7]. Papua Island consists of two provinces, Papua and West Papua. Ethnically, most Papuans are Melanesians belonging to one of six distinct local tribes, with Christianity being the dominant religion. This is in contrast with Java, the most populated island in Indonesia which is populated by Javanese as the most common ethnicity and Indonesia has the largest Muslim in the world [6], [8]. Papua has a special autonomy status due to high revenues from the exploitation of natural resources including gas and oil. Papuans are given privileges in education and health care, which are different from most other provinces in Indonesia [2], [8], [9]. Culturally, Papuans have their local concepts of illness, death, and misfortune which include the widely held belief that death and sickness occur intentionally [8], [10]. The feeling of being stigmatized seems to be common among Papuans. Unfortunately, Papuans are viewed as being primitive in a globalized world [8]. In particular, feeling stigmatized has been found in PLHIV since being infected means breaking cultural norms [11]–[13] and the gap of communication between native Papuan and most migrants healthcare providers might be added being stigmatized among Papuan patients [14].

Despite the status of a special autonomous region, challenges remain to organize health care for Papuans living with HIV because of the large geographical area, the lack of infrastructure and

For peer review only

transportation into many parts, especially the remote highlands. Health care for PLHIV was initially provided by hospitals, but in recent years, is also provided by community health centers [15], [16]. ART is provided free of charge [17]. To ensure the sustainability of the ART supply chains [18]–[20]every PLHIV must be registered in the national cohort before initiation of ART [21]. Even though there were 153 locations which provided HIV care in Papua Province [22], less than 25% of Papuans living with HIV were on ART [7], [22]. The coverage of ART in Papua was lower than other parts of Indonesia [2]. Previous survey-based studies in Papua showed low levels of health literacy, and lower levels of adherence to medication than in other parts of Indonesia. We also found that stigma was significantly associated with non-adherence [23]. To improve the situation for Papuans living with HIV, more in-depth knowledge is needed about the local cultural context explaining the barriers and facilitators to access medication and remain on treatment in the changing health care system.

Objective

This study aimed to explore the perspective and the experiences of Papuans living with HIV and their strategies to take ART.

Methods

Study design, setting and population

This was an exploratory qualitative study. Data were collected between July and September 2018. The study covered all five tribes located in Papua Province, namely LaPago (Wamena), Mee Pago (Timika), both from the highland areas and Anin Ha (Merauke), Saereri (Serui), and Mamta (Jayapura) from the lowland areas. We included patients and HCPs working in HIV care and each site included at least two patients; one male and one female, and two HCPs. The inclusion criteria of patients were: (1) native Papuans or migrants having a Papuan spouse, (2) aged ≥18 years, (3) were on antiretroviral therapy for at least one year before commencing the interview, and (4) willing and able to give informed consent. The inclusion criteria of HCPs

For peer review only

were: (1) working in HIV care for at least two years in a hospital or in a community health center (called *Puskesmas* in Indonesia), (2) having completed training in HIV care, and (3) willing and able to give informed consent.

Study procedure, data collection, and management of data

The interview guide was developed based on literature [24], [25] (Appendix 1). The research team of this study had a diverse background from pharmacy (EIS, EL, KT), epidemiologist (AP), and nursing field (CE) and all researchers are female. Two out five were masters and the remaining was PhD. The interviews conducted by EIS and two research assistants with a background in sociology and nursing who have worked for almost 25 years in HIV care in Jayapura. Interviews were conducted in Bahasa Indonesia. All interviews took place in a private location as agreed between participants and interviewers, and lasted approximately one hour. Topics covered in the interviews were the health problem which triggered getting a test of their HIV status, and the patient's experiences before and after starting to take ART.

We audio-recorded the interviews. The interviewers also took field notes during the interviews. All audio recordings were transcribed verbatim, leaving out noise sounds and conversation outside of the study topics. We limited transcription to 3 interviews per day per transcriber to ensure thoroughness. All transcripts were double-checked. We offered the participants to read the transcripts however all participations did not opt to do so. Each transcript was given a unique identifier composed of the type of participants, gender, and region. No newer codes was found after coding 23 interviews, and we agreed that saturation had been reached for this study. A total codes from PLHIV and HCW were 354 and 299 codes, respectively. From these codes, main themes were extracted and the some main themes identified are presented in appendix. The research assistants received training in ethical practice, and tracking procedures.

Data analysis

For peer review only

Data were analyzed using the Atlas Ti Version 8.4 software. Transcripts were coded by EIS, EL, CE taking a similar approach as in a previous study. A coding framework was developed, once coding was completed, categories were developed. Themes were identified from the categories (Appendix 2). Disagreements in the analysis were resolved by discussions between all authors. The themes were finally mapped within an existing framework [24], [26].

Patient and public involvement

EIS has worked as a HCP in Papua previously and therefore knew HPCs in all facilities. EIS contacted potentially eligible HCPs in all facilities and invited them to participate in the study. HCPs agreed to participate were also asked to contact potentially eligible patients to participate in this study. The participants gave written informed consent before interviews.

Results

Participants

All HCPs who were approached agreed to participate and none of PLHIV were refused to participate this study. Overall, 14 HCPs participated who recruited 13 patients (Table 1). The 14 HCPs were three physicians, nine nurses, one psychologist, and one midwife. The mean age of HCPs was 42 years. Among HCPs, 9 were female and 8 worked in hospital.

Ten out of 13 Papuan patients got their medication from the hospital pharmacy, and the remaining patients accessed *Puskesmas* to collect their ART. The mean age of patients was 33 years. Among the patients, 8 were females, and 11 patients were diagnosed in a Voluntary, Counseling, and Testing (VCT) clinic following ongoing symptoms of illness. Additional participant's characteristic shows in Table 1.

For peer review only

Four themes emerged which were about: (1) organization of care, (2) healthcare provider, (3) social support, and (4) patient's knowledge, beliefs, and routines (Appendix 2).

1. Organization of care

We explored the knowledge of our participants on accessing HIV care including collecting ART in their area. Both patients and HCPs reported *Puskesmas* and hospital had the facilities to diagnose HIV. Some patients had no information about whether they could collect ART in *Puskesmas*. In general, participants revealed that distance and transportation cost were barriers to keep taking ART. Patients felt guilty if they were not able to collect ART.

> *"I had been waiting for the trucks which would bring me to the city to collect my ART. Nevertheless, my boss and the trucks never came, and I heard that there was a problem with gasoline stock supply. That was why I could not get my drugs on time. I blamed myself for that. My ART is my responsibility but I could not say anything since my location was so far." [Patient, Male]*

Even though there is the possibility of collecting ART in *Puskesmas*, this option was rejected by some patients. Patients found the infrastructure and laboratory monitoring insufficient. A community center with only a small room used for all patients irrespective of their disease created fear of being exposed as HIV positive to others.

> *"I think the performance of personnel in Puskemas is good. However, the consultation room is small and without separation. Everyone can hear what nurses are saying… I think everyone can* HCPs *recognize my HIV directly." [Patient, Male]*

Interestingly, HCPs knew that being in full view of their acquaintances and neighbors in *Puskesmas* increased the fear of being identified as HIV positive**.**

> *"I think patients have other opinion why they do not want to access Puskesmas. It is about lifetime medication, they [the patients] must collect their ART routinely. It would raise suspicions from other who might access Puskesmas for other chronic diseases." [HCP, Female]*

For peer review only

Patients weighed the costs and benefits of being treated in *Puskesmas*, some patients preferred the hospital despite having to spend more money and time**.**

> *"I needed about IDR [Indonesian rupiah] 30.000-60.000 every time to collect my ART at a hospital. It may be costlier than collecting ART at Puskesmas, which only take 10.000 IDR, but I decided to keep collecting ART here in hospital." [Patient, Female]*

Patients and HCPs mentioned administration as an important topic. Most patients could not understand why HCPs asked them to show their single identity number (NIK= *Nomor Induk Kependudukan*). The patients thought that this was irrelevant since costs for health care should be covered. One patient expressed her worries about this:

> "M*y concern was only about my card…… My nurses have asked it many times and I could not show it." [Patient, Female]*

Some HCPs explained that patients needed to register with their NIK to be included in the national HIV cohort to receive ART. Without NIK, a delay in starting ART could occur.

Patients and HCPs also discussed the complexity of the administration of the home visit programs. By HCP's perspective that revealed that they needed to attach some pictures as proof to get reimbursement from their health insurance. However, patients felt uncomfortable about the HCPs taking pictures in their homes. Also, patients felt the home visits increased the suspicion in the community. Some patients offered to have meetings with HCPs in another place but not in their house.

> *"I remembered one of my patients refused my visit to his house. He said to me…." please do not come…. I am afraid people will be curious about your visit'. He offered to meet me in other place instead of his house." [HCP, Female]*

Sometimes HCPs found their colleagues documented their activities to make fictitious visits. One HCP shared,

For peer review only

"Most of our colleagues tried making fictitious programs. They made some photos as complementary documents to get reimbursement. Most of our colleagues focused on money and they had many tricks to get reimbursement." [HCP, Female]

The under-utilization of *Puskesmas* has been considered as problems for patients and HCPs. The HCPs were frustrated because they realized that their goal of improving the quality of health services could not be achieved.

"Since I have worked in Puskesmas, I have found the lack of laboratory is still a problem. We want that Puskesmas can be independent and our goal to deliver the high quality of health service can be achieved. I do not know when it is going to happened." [HCP, Female]

2. Healthcare Provider

Patients recognized the competence of HCPs, e.g., to provide information about medications. Patients described the benefits of ART. A patient described why she needed ART for the rest of her life.

'If someone is asking me about ART, I can let them know that ART can suppress my virus but not to cure. I need to take this tablet for a lifetime. My nurses explained this often to me." [Patient, Female]

The language HCPs used was mentioned as an issue. For example, HCPs had problems to express medical terms about side effects in simple words, especially HCPs who were not native Papuans.

"I think language is challenging here. I tried counselling them about side effects, such as rash, or stiffness but I could not find words to replace those into their language. So, sometimes I must say "kaskado" to replace rash." [HCP, Female]

The language gap has also produced misunderstandings in the community, especially in the HIV awareness program. One patient shared his experience about this program before he was diagnosed with HIV as follows.

For peer review only

"The nurses came here often to give some programs about the HIV awareness. They said many things, we listened but we got nothing. We did not understand ...."[Patient, Male]

Some HCPs noted that they often had a deterrent effect when promoting the HIV program in community because they used scary images of skeletons. The concept that HIV could be treated with medication was omitted from the program.

"In early days, we used images of death, skeleton and disfigurement in our HIV awareness program. We hope the community can get a deterrent effect. Later on, the community can learn about how to prevent it. However, we found the information about the HIV syndrome was among the topics most commonly remembered by community. I think most people are stubborn. It is better to use a deterrent effect. We could not say our information about HIV in sweet words. It could not work."[HCP, Female]

"……When we have time to inform about ART that we could not say more such as ART could suppress HIV and could not cure of HIV. Of course, we would like to inform patients and community completely. However, we should be wise to consider our targeted individuals. We say ART is a drug because if we send all information, patients and community could remember only the small part of the end our information." [HCP, Male]

Another issue related to language was to talk about genital terms in prudent ways to patients as this is important for Papuans. Besides verbal language, patients reported about non-verbal signs such as unprofessional behavior of personnel and breaches of confidentiality. One patient strongly articulated his experiences with a HCP.

"A nurse increased her voice when giving us information…it might be her character…. However, I preferred that others who did not have business with my health could not hear" [Patient, Male]

Similarly, HCPs perceived they had power to make patients feel inferior and dependent on health care system without any possibility to protest.

"I felt hurt, when I saw my colleagues treating patients while laughing and sometimes they were busy with their mobile during consultation."[HCP, Female]

For peer review only

One patient revealed her situation when she wanted to ask some information to her health providers

"I kept my mouth suddenly, when I saw her face. I could not open my mouth even I really needed to ask her about my problem." [Patient, Female]

One healthcare provider revealed the breaches of confidentiality occurred in healthcare setting. She also found that the patient preferred to skip medication as a reaction of being disappointed than showing his expressions directly to HCP.

"A few weeks ago, we had problems with our staff. One of our staff was unable to maintain patient's status. He told the status of the patient to the family although the patient asked us to keep it confidential. He was not ready yet to disclose the status. I can understand if he disappeared and did not collect medication for a few months. But everything is now solved." [HCP, Female]

It was commonly found that HCPs refused to treat patients and asked other colleagues to replace their shift in preparing medication.

3. Social support

We found that patients and HCPs had different opinions about social support. In general, a positive relationship with others was described as powerful to support patients over time and reduce the risk of discontinuation of ART.

3.1. Relationship with family

There was both, acceptance and rejection from families. Even though it happened, HCPs considered having family support was essential to be present before they placed patients on ART. The HCPs believed the existence of family would help patients to overcome fear about medication and social problems.

"Most of the patients who had a good relationship with family, they are adherent and there is only a small percentage of them who stopped their ART. However, for patients who hide their

For peer review only

status, most of them found their lives as useless and they could stop ART anytime. The hindrances do not really impact patients when they know their family protect them." [HCP, Female]

Patients revealed different feelings and experiences about the family's support. They expressed that the family influenced the decision to seek health care. There could be a delay in being diagnosed and be on ART because of the family. One patient mentioned her family brought her to hospital which was far away from her house. It was impossible to bring her to the nearest health facility because her father also worked there. In order to receive help from their family, patients needed to disclose their status. To disclose HIV status was described as a double-edged sword, either to receive support or being stigmatized.

"My family know my HIV, they support me to take my medicine, but they have separated my plate, glasses from others."[Patient, Male]

3.2 Relationship with HCPs

In general, the willingness of HCPs to help patients was strongly articulated from both patients and HCPs. For example, HCPs described that patients who had a strong commitment to be adherent on ART might be prioritized. One HCP said,

"If their home is far from the hospital, particularly for patients who fly by plane, we can give them 2 or 3 months of their ART. I appreciated a pregnant woman who travelled by bus from Sarmi [the name of a district] to here. I can see her commitment to prevent her baby from having HIV." [HCP, Female]

Moreover, HCPs revealed there were consequences of their help such as providing more supply of ART to one patient. One HCP said,

"Then after giving the stock for three months, we should be cautious about the stock for other patients. Giving three months ART for patients means we borrow from other patient's stock." [HCP, Female]

One HCP provided other help such as creating a safe environment in the clinic, transportation cost and providing food. One patient agreed that his HCP helped him by providing a safe

environment in clinic as well as giving ART more than one month to reduce him coming often to hospital.

"Here, I feel safe, everybody knows each other and I don't need to hassle and to collect ART, I come here every two months."[Patient, Male]

However, patients also acknowledged the fear of rejection and being stigmatized. One patient explained:

"How they treated me so bad, I must fight in emergency department after they rejected me. I was there not for free. I am Papuan, and I have rights to get health access. Finally, one doctor came then I said to him that I did not want others in that hospital to discriminate more. It was enough." [Patient, Female]

Interestingly, HCPs expressed their willingness to share their private number had been misused by patients. Most of patients contacted them to arrange the place to collect ART out of the hospital instead to discuss problem such as side effects. Most of HCP found patients who accessed hospital had still worries about others.

"Sometimes they were at entrance of the hospital and texted me to bring their tablets to the entrance of the hospital or canteen." [HCP, Female]

3.3 Relationship with peers

Both HCPs and patients held diverse experiences about peers in supporting patients. Some patients described peers to help them to cope with fear and empower them. With peers, patients were not alone anymore and sometimes peers linked them with HIV care better.

"I join peers. I am happy with that. I can meet people who have the same condition with me. We discussed many things, not only medication but how we deal with stress. With them I can discuss many things including something private, ha ha…about our genitals." [Patient, Female]

One patient revealed that a HCP asked her to visit patients who did not attend the hospital for collecting ART.

For peer review only

"In my situation, HCP asked me to visit other patients who did not attend the hospital. HCP considered my visit to patient's house could not raise suspicions. All my expenses to visit other patients were paid by HCP." [Patient, Female]

However, HCPs added the existence of peers was diverse and misinterpretation of patient to response about peer was commonly occurred.

"Sometimes, I was disappointed with some peers. They persuaded my patients who were adherent to move to other health facilities and asked them to try herbal. I know it was not 100% peer's faults, my patients could not filter obtained information but the reason we introduced peers to patients was to help others but to not make others were their followers." [HCP, Female]

4 Patient's knowledge, beliefs and routines

The level of patient's knowledge played a role in coping with HIV. Interestingly, patients who had sufficient knowledge revealed that being healthy and being adherent reduced stigma from others.

"*Many people do not believe that I have HIV and I take my ART. They see me as healthy and might not look like people who lived with HIV. I cannot regret with my past. I must move on." [Patient, Female]*

Differently, a patient who had insufficient knowledge and previously dropped out of care described that they believed in traditional medicine being helpful and believed ART was a not a drug since the it did not cure the symptoms and the disease. A HCP explained:

"The insufficient knowledge is a problem for the patients. I found patients attempting to use traditional medication since they had the lack of knowledge of ART. I think it was because we informed them that their ART is to suppress their virus not to eliminate."[HCP, Female]

A number of difficulties keeping to the regular medication intake schedule were identified. These included being bored or having work-related activities. One patient said,

"I don't think so that my ART is not important, but I must sell 'pinang' (betel nut) every day to support my life and my daughter. I worked until late at night and it made me felt tired and sleepy.

Then I forgot to take my medication. So if I have a break I take my medication directly." [Patient, Female]

Interestingly, most participants took religion seriously. Christian belief shaped attitudes of participants to accept HIV as their disease. Another patient took hope from religious belief that HIV could be cured. They believed that taking ART was the way for God to help them.

"God's miracle will happen, just believe and take ART regularly." [Patient, Male]

In a similar way, HCPs used religion to measure the quality of their service and believed helping patients was the same as serving God.

"I told my colleagues, our salary and incentives would be bloody money, when we were not honest in doing our job. Our money would be like water into our noken (woven bag). It disappears fast because we did not work according to God's will. We are blessed if we treat our patients likely what God wants." [HCP, Female]

In contrast, both patients and HCPs also shared their views that based on the religion patients were immoral, and God used HIV as a warning sign to sinners.

"I think there is a link between HIV and religion. Patients were infected with the virus because they were not afraid to of God's law. They were not married yet, drunk and had sex with women who were not their partner." [HCP, Female]

Discussion

In this qualitative study in Papua province, a region with a high prevalence of HIV and a relatively low uptake of ART, we explored Papuan's experiences of taking ART from the patient and the HCP perspective. We found that organization of care, HCPs, social support and Patient's knowledge, beliefs and routines had an impact on the medication taking of patients.

In line with a previous study we found that just the availability of ART is insufficient for patients to cope with their chronic HIV treatment [27]**.** Our findings are more diverse than a

For peer review only

previous study [24] since we found more factors have an impact on the daily struggles of Papuans in taking ART. Although in recent years, health care has been reorganized, so that HIV care can be provided in remote areas by *Puskesmas*, some patients do not use those because of a lack of privacy. As in other studies, fear of disclosure of HIV status and the stigma associated with disclosure was an important barrier to accept this care closer to home [28]. This was not only found to be a barrier to get supply with ART, but also to discuss concerns and get information on the disease and treatment [27],[29]. Misunderstandings about administrative requirements, possibly unique to the special autonomous status of Papua, also formed a barrier for some patients and seemed to be a source of fraud for HCPs. Contrary to previous study [30], patients valued the competence of HCPs highly. However, both HCPs and patients reported communication barriers, since the local language for example lacks terms to describe side effects with sufficient details. In addition, patients experienced the language and attitude of some HCPs as degrading. Examples included HCPs laughing or not paying attention to patients or discussing sensitive issues like genitals directly and loudly, as have been shown in other studies [8], [31]. Similar as in other studies, use of educational materials producing fear was also perceived to contribute to stigma [9], [32].

Patients and HCPs agreed that support from family, HCP and peers were important for the patients to cope with treatment. In particular the support of the family in medication taking was seen as essential as in other studies [33]–[35]. However despite support, patients could be feeling isolated by their family and family members also feared stigma of the community by disclosing the status of their family member. As in other studies, peer support could be very positive, but also carried the risk of misinforming patients [36]. Furthermore, HCPs experienced difficulties to find sufficient patients who wanted to support their peers because of fear to disclose their status. HCPs recognized that support should be personalized for each patient.

For peer review only

Religion was an important topic to be discussed for patients and HCPs. Contrary to a previous study [36], the religious beliefs empowered patients to take ART and being hopeful. Religious beliefs were also an important motivator for HCPs to offer the best service to patients. However, religious beliefs also contributed to the notion that HIV was a punishment due to personal failure.

Stigma is an effect of HIV and most of PLHIV considered that stigma was more brutal than deadness [37]. Without stigma reduction program is addressed, the existing HIV policies and ART are incomplete to help PLHIV. There is very limited information how to successfully reduce HIV stigma reduction in Indonesia. However, some interventions among people affected with leprosy in Indonesia [38], [39] might be good examples to be implemented. Testimony, and counselling, significantly reduced internal and external stigma among leprosy patients, and the community. Therefore, testimonies of HIV infected women took ART and were successful in having babies with no HIV infection might change the perceptions of community and healthcare providers. Furthermore, strengthening the economic situation of leprosy patients by providing microfinance has been shown to be successful. Similar with previous studies [40], [41], people who were in a better socioeconomic situation had better self-esteem and were actively involved with their community and had less internal stigma.

Strengths and Limitations

It should be noted that there were some limitations in this study. First, we specifically recruited patients from five different tribes in Papua. Other racial/ethnic groups and may have different experiences. Second, we asked HCPs to ask patients to participate in this study, so our results refer to patients who remain in chronic care. More work needs to be done to identify factors

For peer review only

driving patients to drop out of care completely. Despite these limitations, our findings provide new insights into the daily struggle in taking ART among Melanesians in Indonesia.

Conclusion

These data is important for the further development of interventions to support patients in chronic treatment with ART taking into account the specific cultural needs of Papuan HIV patients. In particular, addressing communication barriers is important. The HIV treatment strategies implemented in Papua need to be corrected with some attention to social culture of Papuan. Furthermore, broader strategies to reduce stigma taking into account local culture and religion should be a priority for the centralized and local government after implementation of HIV strategies in Papua has been replicating from the rest of Indonesia area with insufficient interest to social cultural of Papuans. Hopefully, this can improve retention and adherence, and ultimately, health outcomes for Papuans living with HIV.

Competing interests
The authors have indicated that they have no competing interests

Authors' contributions
Conceived and designed the study: EIS, KT, EL, CE, AP. Analyzed the data: EIS, KT, EL, CE, AP. Wrote the paper: EIS, KT, EL, CE, AP. All authors read and approved the final manuscript

Acknowledgements
The authors would like to express their gratitude to Papuans living with HIV and healthcare providers who participated in this study.

Funding
This work was supported by Directorate General of Higher Education (DIKTI) Scholarship provided to EIS. The funding sponsor had no role in the design of study; in the collection, analyses, or interpretation of the results; in writing section, and the decision to publish this.

Ethics and Consent to participate
This study was approved from the Committee on Ethics Universitas Gadjah Mada, Yogyakarta Indonesia (project number: KE/FK/0507/EC/2018).

Patient consent
All participants, patients and healthcare providers, gave written informed consent.

Data sharing statement
The data would not be shared outside of participating research institutions. Any queries on how to access the data set should be to the corresponding author or ira_sianturi@yahoo.co.id

Consent for publication
Not Applicable

For peer review only

For peer review only

References

[1] C. Murray, K. Ortblad, C. Guinovart, and E. Al, “Global, regional, and national incidence and mortality for HIV, tuberculosis, and malaria during 1990–2013: a systematic analysis for the Global Burden of Disease Study 2013,” *Lancet*, vol. 20, no. 384, pp. 1005–1070, 2014.

[2] UN Joint Programme on HIV/AIDS (UNAIDS), “Global AIDS Update: Miles to go—closing gaps, breaking barriers, righting injustices.,” *Report*, p. 268, 2018.

[3] B. L. Genberg, Y. Lee, W. H. Rogers, and I. B. Wilson, “Four types of barriers to adherence of antiretroviral therapy are associated with decreased adherence over time,” *AIDS Behav.*, vol. 19, no. 1, pp. 85–92, 2015.

[4] S. G. Dalmida *et al.*, “Examination of the Role of Religious and Psychosocial Factors in HIV Medication Adherence Rates,” *J. Relig. Health*, vol. 56, no. 6, pp. 2144–2161, 2017.

[5] Anonymous, “Indonesia Demographic and Health Survey 2012,” 2013.

[6] E. N. Arifin, A. Ananta, D. R. W. W. Utami, N. B. Handayani, and A. Pramono, “Quantifying Indonesia’s Ethnic Diversity: Statistics at National, Provincial, and District levels,” *Asian Popul. Stud.*, vol. 11, no. 3, pp. 233–256, 2015.

[7] Dinas Kesehatan Provinsi Papua, “Profil Kesehatan Provinsi Papua Tahun 2016,” 2017.

[8] A. Simonin, J. Bushee, and A. Courcaud, “Social, cultural and political factors in the design of HIV programmes with Papuan highland communities,” *Cult. Health Sex.*, vol. 13, no. sup2, pp. S185–S199, 2011.

[9] L. Butt, “‘Lipstick Girls’ and ‘Fallen Women’: AIDS and Conspiratorial Thinking in Papua, Indonesia,” *Cult. Anthropol.*, vol. 20, no. 3, pp. 412–442, 2005.

[10] L. Butt, G. Numbery, and J. Morin, “The smokescreen of culture: AIDS and the indigenous in Papua, Indonesia.,” *Pac. Health Dialog*, vol. 9, no. 2, pp. 283–9, 2002.

[11] L. Butt, “Local Biologies and HIV/AIDS in Highlands Papua, Indonesia,” *Cult. Med. Psychiatry*, vol. 37, no. 1, pp. 179–194, 2013.

[12] H. M. Kendrick, “Are religion and spirituality barriers or facilitators to treatment for HIV : a systematic review of the literature,” *AIDS Care*, vol. 29, no. 1, pp. 1–13, 2017.

[13] K. Williams, B. G. Haire, S. Nathan, K. Williams, B. G. Haire, and S. Nathan, “‘ They say God punishes people with HIV ’: experiences of stigma and discrimination among adults with HIV in Dili , Timor-Leste,” *Cult. Health Sex.*, vol. 1058, pp. 1–14, 2017.

[14] L. Butt, J. Morin, G. Numbery, I. Eyon, and A. Goo, “Stigma and HIV / AIDS in Highlands Papua,” 2010.

[15] R. Granich, S. Gupta, J. Montaner, B. Williams, and J. M. Zuniga, “Pattern, Determinants, and Impact of HIV Spending on Care and Treatment in 38 High-Burden Low- and Middle-Income Countries,” *J. Int. Assoc. Provid. AIDS Care*, vol. 15, no. 2, pp. 91–100, 2016.

[16] F. M. Ekawati, M. Claramita, K. Hort, J. Furler, S. Licqurish, and J. Gunn, “Patients’

experience of using primary care services in the context of Indonesian universal health coverage reforms," *Asia Pac. Fam. Med.*, vol. 16, no. 1, pp. 1–10, 2017.

[17] Kementerian Kesehatan Republik Indonesia - Direktorat Jenderal Pengendalian Penyakit dan Penyehatan Lingkungan, *Pedoman Nasional Tatalaksana Klinis Infeksi HIV dan Terapi Antiretroviral pada Orang Dewasa*. 2011.

[18] M. Desai, J. W. Rudge, W. Adisasmito, S. Mounier-Jack, and R. Coker, "Critical interactions between Global Fund-supported programmes and health systems: a case study in Indonesia.," *Health Policy Plan.*, 2010.

[19] Shreeshant Prabhakaran, Arin Dutta, Rebecca Ross, and Catherine Barker Cantelmo, "Options To Finance The Rapid Scale-Up Of The HIV Response In Indonesia The Role of the National Health Insurance Scheme (JKN) and Local Governments," 2018.

[20] Anonymous, "Laporan Perkembangan HIV AIDS dan PIMS Triwulan 3 2018.pdf," 2018.

[21] Anonymous (Kementerian Kesehatan Republik Indonesia), *Petunjuk Teknis: Pengisian Formulir Kesehatan Pencatatan dan Pelaporan Program Pengendalian HIV AIDS dan IMS*. 2015.

[22] Kementerian Kesehatan Republik Indonesia, "Laporan Perkembangan HIV-AIDS & PIMS di Indonesia Triwulan 1 tahun 2019," 2019.

[23] S. Rueda *et al.*, "Examining the associations between HIV-related stigma and health outcomes in people living with HIV/AIDS: a series of meta-analyses.," *BMJ Open*, vol. 6, no. 7, p. e011453, 2016.

[24] C. W. Holtzman *et al.*, "Mapping Patient–Identified Barriers and Facilitators to Retention in HIV Care and Antiretroviral Therapy Adherence to Andersen's Behavioral Model," *AIDS Care*, vol. 27, no. 7, pp. 817–828, 2015.

[25] L. Atuyambe, S. Neema, E. Otolok-Tanga, G. Wamuyu-Maina, S. Kasasa, and F. Wabwire-Mangen, "The effects of enhanced access to antiretroviral therapy: A qualitative study of community perceptions in Kampala city, Uganda," *Afr. Health Sci.*, vol. 8, no. 1, pp. 13–19, 2008.

[26] R. M. Anderson, "Revisiting the Behavioral Model and Access to Medical Care : Does it Matter ?," *J. Health Soc. Behav.*, vol. 36, no. 1, pp. 1–10, 1995.

[27] S. Treves-Kagan *et al.*, "Why increasing availability of ART is not enough: A rapid, community-based study on how HIV-related stigma impacts engagement to care in rural South Africa," *BMC Public Health*, vol. 16, no. 1, pp. 1–13, 2016.

[28] F. S. Shabalala *et al.*, "Understanding reasons for discontinued antiretroviral treatment among clients in test and treat: a qualitative study in Swaziland," *J. Int. AIDS Soc.*, vol. 21, p. e25120, 2018.

[29] M. Sugiana, I. N. Sutarsa, and D. P. Duarsa, "Barriers to Integrating Antiretroviral Therapy Services Into Community Health Centre : A Qualitative Study in Badung Regency Hambatan-

For peer review only

hambatan Puskesmas Sebagai Satelit Antiretroviral Therapy (ART) : Penelitian Kualitatif di Kabupaten Badung," *Public Heal. Prev. Med. Arch.*, vol. 3, no. 1, pp. 60–65, 2015.

[30] T. O. Famoroti, L. Fernandes, and S. C. Chima, "Stigmatization of people living with HIV/AIDS by healthcare workers at a tertiary hospital in KwaZulu-Natal, South Africa: A cross-sectional descriptive study," *BMC Med. Ethics*, vol. 14, no. SUPPL.1, pp. 1–10, 2013.

[31] F. van Loggerenberg *et al.*, "A Qualitative Study of Patient Motivation to Adhere to Combination Antiretroviral Therapy in South Africa," *AIDS Patient Care STDS*, vol. 29, no. 5, pp. 299–306, 2015.

[32] M. Flassy, "Deficit of Women ' s Human Right in Papua Province : A Conceptual Framework," vol. 2, no. 2, pp. 28–52, 2019.

[33] S. P. Wasti *et al.*, "Factors influencing adherence to antiretroviral treatment in Asian developing countries: A systematic review," *Trop. Med. Int. Heal.*, vol. 17, no. 1, pp. 71–81, 2012.

[34] J. Kibicho and J. Owczarzak, "A Patient-Centered Pharmacy Services Model of HIV Patient Care in Community Pharmacy Settings: A Theoretical and Empirical Framework," *AIDS Patient Care STDS*, vol. 26, no. 1, pp. 20–28, 2011.

[35] J. F. Xu, Z. Q. Ming, Y. Q. Zhang, P. C. Wang, J. Jing, and F. Cheng, "Family support, discrimination, and quality of life among ART-treated HIV-infected patients: A two-year study in China," *Infect. Dis. Poverty*, vol. 6, no. 1, pp. 1–10, 2017.

[36] S. Kulkarni *et al.*, "Identifying Perceived Barriers along the HIV Care Continuum: Findings from Providers, Peer Educators, and Observations of Provider–Patient Interactions in Ethiopia," *J Int. Assoc Provid AIDS Care*, vol. 15, no. 4, pp. 291–300, 2016.

[37] H. A. Cross, M. Heijnders, A. Dalal, S. Sermrittirong, and S. Mak, "Interventions for stigma reduction-part 2: Practical applications," *Asia Pacific Disabil. Rehabil. J.*, vol. 22, no. 3, pp. 71–80, 2011.

[38] R. M. H. Peters, Dadun, M. B. M. Zweekhorst, J. F. G. Bunders, Irwanto, and W. H. van Brakel, "A Cluster-Randomized Controlled Intervention Study to Assess the Effect of a Contact Intervention in Reducing Leprosy-Related Stigma in Indonesia," *PLoS Negl. Trop. Dis.*, vol. 9, no. 10, pp. 1–24, 2015.

[39] M. Lusli *et al.*, "The Impact of a Rights-Based Counselling Intervention to Reduce Stigma in People Affected by Leprosy in Indonesia," *PLoS Negl. Trop. Dis.*, vol. 10, no. 12, pp. 1–25, 2016.

[40] D. Dadun *et al.*, "Impact of socio-economic development, contact and peer counselling on stigma against persons affected by leprosy in Cirebon, Indonesia - a randomised controlled trial," *Lepr. Rev.*, vol. 88, no. 1, pp. 2–22, 2017.

[41] D. Dadun, R. M. H. Peters, W. H. van Brakel, J. G. F. Bunders, I. Irwanto, and B. J. Regeer, "Assessing the impact of the twin track socio-economic intervention on reducing leprosy-

For peer review only

related stigma in Cirebon District, Indonesia," *Int. J. Environ. Res. Public Health*, vol. 16, no. 3, pp. 1–20, 2019.

For peer review only

Table 1. Characteristics of study participants (n=27)

Participant	Characteristics	Number (%)	*Mamta (Jayapura)*	*Mee Pago (Timika)*	*Saereri (Serui)*	*Anin Ha (Merauke)*	*LaPago (Wamena)*
Healthcare providers (n=14)	Mean age 42 (range:35-55 years)						
	Work at Hospital	8 (57%)	1	1	3	2	1
	Work at Community health center (*Puskesmas*)	6 (43%)	2	2	1	0	1
	Female	9 (64%)	1	3	2	1	2
	Male	5 (36%)	2	0	2	1	0
	Physician	3 (12%)	1	0	1	1	0
	Midwife	1 (7%)	0	0	1	0	0
	Nurse	9 (64%)	2	3	1	1	2
	Psychologist	1 (7%)	0	0	1	0	0
Patients (n=13)	Mean age 33 (range:20-60 years)						
	Medication dispensed from hospital pharmacy	10 (77%)	3	0	3	3	1
	Medication dispensed from community health center (*Puskesmas*)	3 (23%)	0	2	0	0	1
	Female	8 (61%)	2	1	2	2	1
	Male	5 (39%)	1	1	1	1	1
	Diagnosis following VCT	11 (85%)	3	2	2	3	1
	Diagnosis on antenatal ward	2 (15%)	0	0	1	0	1

VCT: Voluntary, Counselling, and Testing; *Puskesmas*: *Pusat Kesehatan Masyarakat*

For peer review only

Appendix 1 Interview Guide

Main questions:

1. What were the health problems forcing you to test your HIV status?

2. What were your experience before and after HCPs placed you on ART?

Probing Question (not necessarily being asked, depends on answer from the main question):

1. *How long you have been initiated ART since you were diagnosed with HIV?*
2. *How convenient is it to get ART in this facility?*
3. *Have you planned to access another facility?*
4. *In your view, do you think HIV/AIDS is still a big threat in this community?*
5. *Where do most HIV/AIDS patients seek care?*
6. *What type of care is available for HIV/AIDS patients in this community?*
7. *What sort of treatment is usually given to HIV/AIDS patients in this area?*
8. *What are some of the beliefs regarding risk to HIV infection with increased availability of ART?*
9. *What prevention measures against HIV/AIDS do people in this area use (probe for condom use, reducing sexual partners, abstinence, etc.)*
10. *What is the community's attitude towards HIV prevention with availability of ART for AIDS treatment?*

For peer review only

Appendix 2 Summary of Qualitative Results

Theme	Descriptions	Category	Patient's Illustrative quotes	HCP's Illustrative quotes
Organization of care	This theme is to investigate whether participants especially patients understand type of health facility which provide HIV care. This theme is to find patient's preference for selecting the existing health facility in this area.	-Small consultation room (B)<br>-Insufficiency laboratory (B) monitoring<br>-Complex administration (B)<br>-Being in full view of their acquaintances and neighbors (B)	*"My condition was checked by nurse, I have never met doctors here. I think health service in Jayapura is better than here." [Male]*	*"I can show you that some dipstics are not available here then we had problem to bring our service optimal to our patients." [Male]*
Healthcare Provider	This theme associates to the ideal criteria for HCP working in the HIV area	-Knowledge (B/F)<br>-Language (B/F)<br>-The unprofessional behavior of personnel and breaches of confidentiality (B)	*"I tried to ask the nurses, but when I was looking at her, she was not antistatic with me. I was scary and I could not say anything about my problem to her." [Female]*	*"The doctors are egoist, Their words can kill people. If I told them to more concern on patients by seeing lab results then they would say…. "You should study medicine so you can be doctors not like this". Can you imagine they could say like this to colleagues and how about the patients?" [Female]*

For peer review only

Social support	This theme is to collect information whether patients and HCPs considered support must be available during patients on ART. This theme is to find the type of support which are available	-Family (B/F)<br>-Healthcare Providers (B/F)<br>-Peer (B/F)	*"I think my family is everything, without their supports I think I could stand here. Without them, I think I was death." [Female]*	*"It is common their family came here to collect their ART, one patients have relative in administration ward, she is almost every month came here to collect ART in helping family." [Female]*<br><br>*"But suddenly patients came and asked me to refer them to another health facility because I knew his peer also moved to that facility." [Female]*
Patient's knowledge, beliefs and routines	This theme is about barriers or facilitators of participant to be able to be adherent based on their personal knowledge, beliefs and routines.	-Being bored, forget, busy with working (B)<br>-Economic factor, transportation cost(B)<br>-Religion (B/F)	*"I have problem to remember my schedule, I must work until late, if my stuffs are sold, I can go back at 8 o'clock, so I have time to take my medication at 10 o'clock." [Female]*<br><br>"*My parents and I are surrender to God because He is powerful, everything is possible for Him." [Male]*	*"Yes, side effects is commonly occurred, but I have never heard patients stopped taking ART because of side effects. My nurses have already reminded me about possibility of headache after taking ART. But they said it was adjustment period, after" not more than 6 weeks all complain will be gone….."[Female]*

B: Barrier
F: Facilitator

For peer review only

Consolidated criteria for reporting qualitative studies (COREQ): 32-item checklist

No. Item	Guide questions/description	Reported on Page #	Checklists
**Domain 1: Research team and reflexivity**			
Personal Characteristics			
1. Inter viewer/facilitator	Which author/s conducted the inter view or focus group?	Methods	√
2. Credentials	What were the researcher's credentials? E.g. PhD, MD	Methods	√
3. Occupation	What was their occupation at the time of the study?	Methods	√
4. Gender	Was the researcher male or female?	Methods	√
5. Experience and training	What experience or training did the researcher have?	Methods	√
Relationship with participants			
6. Relationship established	Was a relationship established prior to study commencement?	N/A	
7. Participant knowledge of the interviewer	What did the participants know about the researcher? e.g. personal goals, reasons for doing the research	Methods	√
8. Interviewer characteristics	What characteristics were reported about the inter viewer/facilitator? e.g. Bias, assumptions, reasons and interests in the research topic	Methods	√
**Domain 2: study design**			
Theoretical framework			
9. Methodological orientation and Theory	What methodological orientation was stated to underpin the study? e.g. grounded theory, discourse analysis, ethnography, phenomenology, content analysis	Methods	√
Participant selection			
10. Sampling	How were participants selected? e.g. purposive, convenience, consecutive, snowball	Methods	√
11. Method of approach	How were participants approached? e.g. face-to-face, telephone, mail, email	Methods	√
12. Sample size	How many participants were in the	Results	√

For peer review only

	study?		
13. Non-participation	How many people refused to participate or dropped out? Reasons?	Results	√
Setting			
14. Setting of data collection	Where was the data collected? e.g. home, clinic, workplace	Methods	√
15. Presence of non-participants	Was anyone else present besides the participants and researchers?	Methods	√
16. Description of sample	What are the important characteristics of the sample? e.g. demographic data, date	Methods and Results	√
Data collection			
17. Interview guide	Were questions, prompts, guides provided by the authors? Was it pilot tested?	Methods	√
18. Repeat interviews	Were repeat inter views carried out? If yes, how many?	N/A	
19. Audio/visual recording	Did the research use audio or visual recording to collect the data?	Methods	√
20. Field notes	Were field notes made during and/or after the inter view or focus group?	Methods	√
21. Duration	What was the duration of the inter views or focus group?	Methods	√
22. Data saturation	Was data saturation discussed?	Methods	√
23. Transcripts returned	Were transcripts returned to participants for comment and/or correction?	Methods	√
**Domain 3: analysis and findings**			
Data analysis			
24. Number of data coders	How many data coders coded the data?	Methods	√
25. Description of the coding tree	Did authors provide a description of the coding tree?	Appendix	√
26. Derivation of themes	Were themes identified in advance or derived from the data?	Methods and appendix	√
27. Software	What software, if applicable, was used to manage the data?	Atlas Ti	√
28. Participant checking	Did participants provide feedback on the findings?	N/A	
Reporting			
29. Quotations presented	Were participant quotations presented to illustrate the themes/findings? Was each quotation identified? e.g. participant number	Results	√
30. Data and findings consistent	Was there consistency between the data presented and the findings?	N/A	

For peer review only

31. Clarity of major themes	Were major themes clearly presented in the findings?	Results	√
32. Clarity of minor themes	Is there a description of diverse cases or discussion of minor themes?	Discussion	√

Once you have completed this checklist, please save a copy and upload it as part of your submission. When requested to do so as part of the upload process, please select the file type: *Checklist*. You will NOT be able to proceed with submission unless the checklist has been uploaded. Please DO NOT include this checklist as part of the main manuscript document. It must be uploaded as a separate file.

For peer review only

BMJ Open

The daily struggle to take antiretrovirals: a qualitative study in Papuans living with HIV and their healthcare providers

Journal:	*BMJ Open*
Manuscript ID	bmjopen-2020-036832.R1
Article Type:	Original research
Date Submitted by the Author:	17-May-2020
Complete List of Authors:	Sianturi, Elfride; Groningen University Faculty of Mathematics and Natural Sciences, Groningen Research Institute of Pharmacy Unit PharmacoTherapy, -Epidemiology & -Economics (PTEE); Universitas Cenderawasih,<br>Latifah, E; Universitas Muhammadiyah Magelang<br>Probandari, Ari; Universitas Sebelas Maret, Department of Public Health, Faculty of Medicine<br>Effendy, C; Gadjah Mada University Faculty of Medicine<br>Taxis, Katja; Groningen University Faculty of Mathematics and Natural Sciences, Groningen Research Institute of Pharmacy Unit PharmacoTherapy, -Epidemiology & -Economics (PTEE)
**Primary Subject Heading**:	Qualitative research
Secondary Subject Heading:	Health policy, Public health, Sociology
Keywords:	HIV & AIDS < INFECTIOUS DISEASES, Quality in health care < HEALTH SERVICES ADMINISTRATION & MANAGEMENT, EPIDEMIOLOGY, Health policy < HEALTH SERVICES ADMINISTRATION & MANAGEMENT, PRIMARY CARE

SCHOLARONE™
Manuscripts

I, the Submitting Author has the right to grant and does grant on behalf of all authors of the Work (as defined in the below author licence), an exclusive licence and/or a non-exclusive licence for contributions from authors who are: i) UK Crown employees; ii) where BMJ has agreed a CC-BY licence shall apply, and/or iii) in accordance with the terms applicable for US Federal Government officers or employees acting as part of their official duties; on a worldwide, perpetual, irrevocable, royalty-free basis to BMJ Publishing Group Ltd ("BMJ") its licensees and where the relevant Journal is co-owned by BMJ to the co-owners of the Journal, to publish the Work in this journal and any other BMJ products and to exploit all rights, as set out in our licence.

The Submitting Author accepts and understands that any supply made under these terms is made by BMJ to the Submitting Author unless you are acting as an employee on behalf of your employer or a postgraduate student of an affiliated institution which is paying any applicable article publishing charge ("APC") for Open Access articles. Where the Submitting Author wishes to make the Work available on an Open Access basis (and intends to pay the relevant APC), the terms of reuse of such Open Access shall be governed by a Creative Commons licence – details of these licences and which Creative Commons licence will apply to this Work are set out in our licence referred to above.

Other than as permitted in any relevant BMJ Author's Self Archiving Policies, I confirm this Work has not been accepted for publication elsewhere, is not being considered for publication elsewhere and does not duplicate material already published. I confirm all authors consent to publication of this Work and authorise the granting of this licence.

For peer review only

The daily struggle to take antiretrovirals: a qualitative study in Papuans living with HIV and their healthcare providers

E.I Sianturi[1,2,] E. Latifah[3], A. Probandari[4], C. Effendy[5], K. Taxis[1]

[1]PharmacoTherapy,-Epidemiology&-Economics (PTEE) Department of Pharmacy, University of Groningen, the Netherlands

[2]Faculty of Mathematics and Natural Sciences University of Cenderawasih, Papua-Indonesia

[3]Department of Pharmacy, Faculty of Health Science, Universitas Muhammadiyah Magelang, Magelang, Indonesia

[4]Department of Public Health, Faculty of Medicine, Universitas Sebelas Maret, Jl. Ir. Sutami 36A, Surakarta, 57126, Indonesia.

[5]School of Nursing, Faculty of Medicine, Public Health and Nursing, Universitas Gadjah Mada, Yogyakarta, Indonesia.

Correspondence should be addressed to:

E.I Sianturi, Antonius Deusinglaan 1, 9713 AV, Groningen, The Netherlands, Contact Phone Number: +31 0622820392, Fax number: +31 503637576; Contact email: e.i.sianturi@rug.nl; ira_sianturi@yahoo.co.id

Email's authors:

E.I Sianturi: ira_sianturi@yahoo.co.id; e.i.sianturi@rug.nl

E. Latifah: elmiawatilatifah@ummgl.ac.id

A. Probandari: ariprobandari@yahoo.com

C. Effendy: christantie@ugm.ac.id

K. Taxis: k.taxis@rug.nl

For peer review only - http://bmjopen.bmj.com/site/about/guidelines.xhtml

ABSTRACT

Objective: The aim of the study was to explore the experiences of Papuans living with HIV to take Antiretroviral Therapy (ART) from the patient and the health care providers (HCPs) perspective.

Design: This was a qualitative study covering all five tribes located in Papua Provinces one of two Indonesian provinces on Papua Island. Semi structured interviews were conducted with Papuans living with HIV and their HCPs. Interviews were transcribed verbatim and coded to find themes.

Results: Overall, we conducted interviews with 13 Papuans living with HIV (mean age: 33 years, 61 % female) and 14 HCPs (mean age: 42 years, 64% female) within five customary areas. HCPs included three physicians, nine nurses, two others. Two main themes were identified: (1) personal factors, and (2) healthcare environment-related factors. Personal factors were beliefs and knowledge of ART, stigma from family, community, and HCPs as well as practical problems such as transportation because of long distance. Within the theme of healthcare environment, the competences and attitudes of HCPs were particularly relevant. The findings are important in refining HIV treatment strategies implemented in Papua, especially when extending HIV care provided by community centers.

Conclusions: Despite free access to ART, Papuans living with HIV struggle to remain on treatment. Considering local culture and religion in strategies to reduce stigma should be a priority.

Keyword: HIV, Papuan, Melanesian, tribes, customary-area, local culture, stigma, Indonesia

For peer review only

Strengths and Limitations of this study

- The findings provide new insights into the daily struggle in taking ART among Melanesians in Indonesia.
- This study is the basis for quantitative studies to identify how widespread some of the issues are and the findings should inspire others to explore the local context which is important to know to develop strategies to strengthen primary health care in many parts of the world.
- This study specifically recruited patients from five different tribes in Papua. Other racial/ethnic groups may have different experiences.
- Recruiting patients through their health care professionals may create the risk that patients feel coerced to participate in the study.

For peer review only - http://bmjopen.bmj.com/site/about/guidelines.xhtml

BACKGROUND

Antiretroviral therapy (ART) has saved millions of people living with HIV (PLHIV) [1]. Treatment is lifelong. Despite such successes, access to treatment and retention in care is still an issue in many parts of the world [2]. Economic barriers, stigma, social relationships, religion, local concepts about illness and medication impact on the care for PLHIV [3], [4].

Indonesia consists of more than 17 thousand islands and has been viewed as one of Southeast Asia's highest performing economies in recent decades [5]. A large number of islands has fostered the development of a diverse culture with more than four hundred ethnic groups, each with their own language(s) [5], [6]. The prevalence of HIV is about 0.1% across Indonesia. The prevalence in Papua is about 2.3%, with the highest prevalence found in the remote areas of the highlands [7]. Papua Island consists of two provinces, Papua and West Papua. Ethnically, most Papuans are Melanesians belonging to one of six distinct local tribes, with Christianity being the dominant religion. This is in contrast to the rest of Indonesia where Islam is the dominant religion. Within Indonesia, Papua has a special autonomy status due to high revenues from the exploitation of natural resources [2], [8], [9]. In many instances, Papuans are viewed as being primitive in a globalized world [8]. The feeling of being stigmatized seems to be common among Papuans and it has been growing due to inequalities and poor security levels [10]. In particular, feeling stigmatized has been found in PLHIV since being infected means breaking cultural norms [11]–[13]. A prior study showed that stigma among female Papuans with HIV was even more pronounced than male Papuans [14].

Despite the status of a special autonomous region, challenges remain to organize health care for Papuans living with HIV because of the large geographical area, the lack of infrastructure and transportation into many parts, especially the remote highlands. Other challenges include the high number of illiterates, especially in the remote areas [15] and the local concepts of illness, death, and misfortune which include the widely held belief that death and sickness occur intentionally [8], [15]. Such cultural concepts may result in communication barriers between health care workers who are mainly migrants from other areas of Indonesia and the native Papuans [16]. Health care for PLHIV was initially provided by hospitals, but in recent years, is also provided by community health centers [16], [17].

ART is provided free of charge in Papua [18]. To ensure the sustainability of the ART supply chain [19]–[21], every PLHIV must be registered in the national cohort before initiation of ART

For peer review only - http://bmjopen.bmj.com/site/about/guidelines.xhtml

[22]. Even though there were 153 locations which provided HIV care in Papua Province [23], less than 25% of Papuans living with HIV were on ART [7], [23]. The coverage of ART in Papua was lower than in other parts of Indonesia [2]. Previous survey-based studies in Papua showed low levels of health literacy, and lower levels of adherence to medication than in other parts of Indonesia. We also found that enacted stigma was significantly associated with non-adherence among Papuans [24]. To improve the situation for Papuans living with HIV, more in-depth knowledge is needed on barriers and facilitators to access medication and remain on treatment in particular in the changing organization of health care taking into account the local culture. The aim of the study was to explore the experiences of Papuans living with HIV to take ART from the patient and the HCP perspective.

METHODS

Study design, setting and population

This was an exploratory qualitative study. Data were collected between July and September 2018. The study covered all five customary areas in Papua Province, namely LaPago (Wamena), Mee Pago (Timika), both from the highland areas and Anin Ha (Merauke), Saereri (Serui), and Mamta (Jayapura) from the lowland areas. We included patients who received ART either from a hospital or from a community health center, and HCPs working in HIV care. Each site included at least two patients; one male and one female, and two HCPs. The inclusion criteria of patients were: (1) native Papuans or migrants having a Papuan spouse, (2) aged ≥18 years, (3) were on antiretroviral therapy for at least one year before commencing the interview, and (4) willing and able to give informed consent. The inclusion criteria of HCPs were: (1) working in HIV care for at least two years in a hospital or in a community health center (called *Puskesmas* in Indonesia), (2) having completed training in HIV care, and (3) willing and able to give informed consent. The study was approved by the Ethics Commission, Faculty of Medicine, Public Health, and Nursing Universitas Gadjah Mada (number: KE/FK/0507/EC/2018). Before the interviews, participants were informed about the study purposes and ethical approval and signed consent was obtained from each participant. We assured all participants that data would be kept confidential and participants would have no disadvantages in receiving health care.

Study procedure, data collection, and management of data

The interview guide was developed based on literature [25], [26] (Appendix). The research team of this study had a diverse background from pharmacy (EIS, EL, KT), epidemiologist

For peer review only - http://bmjopen.bmj.com/site/about/guidelines.xhtml

(AP), and nursing field (CE) and all researchers are female. Two with a masters degree and three with a PhD degree. The interviews were conducted by EIS and two research assistants with a background in sociology and nursing who have worked for almost 25 years in HIV care in Jayapura. Interviews were conducted in Bahasa Indonesia which is the national language of Indonesia. All interviews took place in a private location as agreed between participants and interviewers, and lasted approximately one hour. Topics covered in the interviews were the health problems which triggered getting a test of their HIV status, and the patient's experiences before and after starting to take ART.

We audio-recorded the interviews. The interviewers also took field notes during the interviews. All audio recordings were transcribed verbatim, leaving out noise sounds and conversation outside of the study topics. We limited transcription to 3 interviews per day per transcriber to ensure thoroughness. All transcripts were double-checked. We offered the participants to read the transcripts, but none of the participations wished to do so. Each transcript was given a unique identifier composed of the type of participant, gender, and region.

Data analysis

Data were analyzed using the Atlas Ti Version 8.4 software. Transcripts were coded by EIS, EL, CE taking a similar approach as in a previous study. We chose a content-oriented approach to analyse the data (undirected coding). All transcripts were read and re-read to develop an initial coding framework which was refined during coding. Once the coding was completed, categories were developed. Themes were identified from the categories. Disagreements in the analysis were resolved by discussions between all authors. The themes were finally mapped with an existing larger framework [25], [27].

Patient and public involvement

EIS has worked as a HCP in Papua previously and therefore knew HPCs in all facilities. EIS contacted potentially eligible HCPs in all facilities and invited them to participate in the study. HCPs agreed to participate were also asked to contact potentially eligible patients to participate in this study. The participants gave written informed consent before interviews.

For peer review only - http://bmjopen.bmj.com/site/about/guidelines.xhtml

RESULTS

Participants

All HCPs and PLHIV who were approached agreed to participate in the study. Overall, 14 HCPs participated who recruited 13 patients (Table 1). The 14 HCPs were three physicians, nine nurses, one psychologist, and one midwife. The mean age of HCPs was 42 years. Among HCPs, 9 were female and 8 worked in hospital. Ten out of 13 Papuan patients got their medication from the hospital pharmacy, and the remaining patients accessed *Puskesmas* to collect their ART. The mean age of patients was 33 years. Among the patients, 8 were females, and 11 patients were diagnosed in a Voluntary, Counseling, and Testing (VCT) clinic following ongoing symptoms of illness. Additional participant's characteristic shows in Table 1.

The codes and categories were mapped on the two themes from existing frameworks [25],[27]. The themes were: (1) factors that represented patient's personal experiences of taking ART and (2) the descriptions of the healthcare environment which played a role in taking ART.

Theme 1: Patient Factors

We identified personal beliefs and knowledge about ART, religion, verbal and nonverbal communication, finances and transport, social support, and other responsibilities as being important factors for patients in dealing with taking ART.

Personal beliefs and knowledge about ART

A patient who previously dropped out of care described that they believed in traditional medicine being helpful. The patient also believed ART was a not a drug since ART did not cure the symptoms and the disease.

> *"My doctor said that my ART required a lifetime commitment. Then I thought ART was just to extend my life not for making my virus disappear…"[Patient, Male]*

An HCP explained insufficient knowledge has challenged patients to try alternative medication.

> *"The insufficient knowledge is a problem for the patients. I found patients attempting to use traditional medication since they had the lack of knowledge of ART. I think it was because we informed them that their ART is to suppress their virus not to eliminate. "[HCP, Female]*

For peer review only - http://bmjopen.bmj.com/site/about/guidelines.xhtml

Differently, the patient's knowledge level played a role in coping with HIV.

Religion

Most participants took religion seriously. Christian belief shaped attitudes of participants to accept HIV as their disease. Another patient took hope from religious belief that HIV could be cured. They believed that taking ART was the way for God to help them.

> *" God's miracle will happen, just believe and take ART regularly." [Patient, Male]*

In a similar way, HCPs used religion to measure the quality of their service and believed helping patients was the same as serving God.

> *"I told my colleagues, our salary and incentives would be bloody money, when we were not honest in doing our job. Our money would be like water into our noken (woven bag). It disappears fast because we did not work according to God's will. We are blessed if we treat our patients likely what God wants." [HCP, Female]*

In contrast, both patients and HCPs also shared their views that based on the religion patients were immoral, and God used HIV as a warning sign to sinners.

> *"I think there is a link between HIV and religion. Patients were infected with the virus because they were not afraid to of God's law. They were not married yet, drunk and had sex with women who were not their partner." [HCP, Female]*

Verbal and nonverbal communication

Participants stressed it was important to talk about genital terms in prudent ways as this is important for Papuans. Besides verbal language, patients reported about non-verbal signs such as unprofessional behavior of personnel and breaches of confidentiality. One patient strongly articulated his experiences with a HCP.

> *"A nurse increased her voice when giving us information…it might be her character…. However, I preferred that others who did not have business with my health could not hear" [Patient, Male]*

The language HCPs used was mentioned as an issue. For example, HCPs had problems to express medical terms about side effects in simple words, especially HCPs who were not native Papuans.

For peer review only - http://bmjopen.bmj.com/site/about/guidelines.xhtml

"I think language is challenging here. I tried counselling them about side effects, such as rash, or stiffness but I could not find words to replace those into their language. So, sometimes I must say "kaskado" to replace rash." [HCP, Female]

The gap in language has also produced misunderstandings in the community, especially in the HIV awareness program. One patient shared his experience about this program before he was diagnosed with HIV as follows.

"The nurses came here often to give some programs about the HIV awareness. They said many things, we listened but we got nothing. We did not understand …."[Patient, Male]

Finance and transport

Patients weighed the costs and benefits of being treated in *Puskesmas* however some patients preferred the hospital despite having to spend more money and time.

"I needed about IDR [Indonesian rupiah] 30.000-60.000 every time to collect my ART at a hospital. It may be costlier than collecting ART at Puskesmas, which only take 10.000 IDR, but I decided to keep collecting ART here in hospital." [Patient, Female]

In general, participants said that distance and transportation cost were barriers to keep taking ART.

"I had been waiting for the trucks which would bring me to the city to collect my ART. Nevertheless, my boss and the trucks never came, and I heard that there was a problem with gasoline stock supply." [Patient, Male]

Social support from family and peers

We found that patients and HCPs had different opinions about social support. In general, a positive relationship with others was described as powerful to support patients over time and reduce the risk of discontinuation of ART. There was both, acceptance and rejection from families. Even though it happened, HCPs considered having family support was essential to be present before they placed patients on ART. The HCPs believed the existence of family would help patients to overcome fear about medication and social problems.

"Most of the patients who had a good relationship with family, they are adherent and there is only a small percentage of them who stopped their ART. However, for patients who hide their status, most of them found their lives as useless and they could stop ART

For peer review only - http://bmjopen.bmj.com/site/about/guidelines.xhtml

anytime. The hindrances do not really impact patients when they know their family protect them." [HCP, Female]

Patients revealed different feelings and experiences about the family's support. They expressed that the family influenced the decision to seek health care. There could be a delay in being diagnosed and be on ART because of the family. One patient mentioned her family brought her to hospital, which was far away from her house. It was impossible to bring her to the nearest health facility because her father also worked there.

In order to receive help from their family, patients needed to disclose their status. To disclose HIV status was described as a double-edged sword, either to receive support or being stigmatized.

"My family know my HIV, they support me to take my medicine, but they have separated my plate, glasses from others."[Patient, Male]

Participants also reported about their experiences with support from peers, i.e. other PLHIV. In some locations formal or informal peer-support groups exist. Some patients described that knowing peers helped them to cope with fear and empower them. With peers, patients were not alone anymore and sometimes peers linked them with better HIV care.

"I join peers. I am happy with that. I can meet people who have the same condition with me. We discussed many things, not only medication but how we deal with stress. With them I can discuss many things including something private, ha ha…about our genitals." [Patient, Female]

One patient revealed that a HCP asked her to visit patients who did not attend the hospital for collecting ART.

"In my situation, HCP asked me to visit other patients who did not attend the hospital. HCP considered my visit to patient's house could not raise suspicions. All my expenses to visit other patients were paid by HCP." [Patient, Female]

HCPs said that the influence of peers may be positive or negative.

"Sometimes, I was disappointed with some peers. They persuaded my patients who were adherent to move to other health facilities and asked them to try herbals. I know it was not 100% peer's faults, my patients could not filter obtained information but the reason

For peer review only - http://bmjopen.bmj.com/site/about/guidelines.xhtml

we introduced peers to patients was to help others but to not make others their followers." [HCP, Female]

Other responsibilities

A number of difficulties keeping to the regular medication intake schedule were identified. These included being bored or having other responsibilities, e.g., work-related activities which made it difficult to take ART. One patient said,

"I don't think so that my ART is not important, but I must sell 'pinang' (betel nut) every day to support my life and my daughter. I worked until late at night and it made me felt tired and sleepy. Then I forgot to take my medication. So if I have a break I take my medication directly." [Patient, Female]

Theme 2: Health Care Environment

Within the theme of health care environment, we identified health service and health care providers as important factors which influenced the experiences of patients in taking ART. Infrastructure, and perceived complicated administrative system were most commonly reported as health service-related factors. Participants also shared their experiences with the home visit programme and educational services. Provider factors consisted of willingness to help, competence of HCPs and trust in HCP..

Health service factors

Infrastructure

Both patients and HCPs reported *Puskesmas* and hospital had the facilities to diagnose HIV. Some patients did not know that it was possible to collect ART in *Puskesmas*. Even though there is the possibility of collecting ART in *Puskesmas*, this option was rejected by some patients. Patients found the infrastructure and laboratory monitoring insufficient. A community center with only a small room used for all patients irrespective of their disease created fear of being exposed as HIV positive to others.

"I think the performance of personnel in Puskemas is good. However, the consultation room is small and without separation. Everyone can hear what nurses are saying… I think everyone can HCPs *recognize my HIV directly.*" *[Patient, Male]*

The under-utilization of *Puskesmas* has been considered as problems for patients and HCPs. The HCPs were frustrated because they realized that their goal of improving the quality of health services could not be achieved.

For peer review only - http://bmjopen.bmj.com/site/about/guidelines.xhtml

> *"Since I have worked in Puskesmas, I have found the lack of laboratory is still a problem. We want that Puskesmas can be independent and our goal to deliver the high quality of health service can be achieved. I do not know when it is going to happen." [HCP, Female]*

Perceived complicated administration system

Patients and HCPs mentioned administration as an important topic. Most patients could not understand why HCPs asked them to show their single identity number (NIK= *Nomor Induk Kependudukan*). The patients thought that this was irrelevant since costs for health care should be covered. One patient expressed her worries about this:

> "M*y concern was only about my card…… My nurses have asked it many times and I could not show it." [Patient, Female]*

Some HCPs explained that patients needed to register with their NIK to be included in the national HIV cohort to receive ART. Without NIK, a delay in starting ART could occur.

Home visit programme and educational services

Patients and HCPs also discussed the effectiveness of the home visit programs. HCP's perspective revealed that they needed to attach some pictures as proof to get reimbursement from their health insurance. However, patients felt uncomfortable about the HCPs taking pictures in their homes. Also, patients felt the home visits increased the suspicion in the community. Some patients offered to have meetings with HCPs in another place but not in their house.

> *"I remembered one of my patients refused my visit to his house. He said to me…." please do not come…. I am afraid people will be curious about your visit'. He offered to meet me in other place instead of his house." [HCP, Female]*

Sometimes HCPs found their colleagues documented their activities to make fictitious visits. One HCP shared,

> *"Most of our colleagues tried making fictitious programs. They made some photos as complementary documents to get reimbursement. Most of our colleagues focused on money and they had many tricks to get reimbursement." [HCP, Female]*

For peer review only - http://bmjopen.bmj.com/site/about/guidelines.xhtml

Some HCPs noted that they often had a deterrent effect when promoting the HIV program in community because they used scary images of skeletons. The concept of HIV could be treated with medication was omitted from the program.

> *"In early days, we used images of death, skeleton and disfigurement in our HIV awareness program. We hope the community can get a deterrent effect. Later on, the community can learn about how to prevent it. However, we found the information about the HIV syndrome was among the topics most commonly remembered by community. I think most people are stubborn. It is better to use a deterrent effect. We could not say our information about HIV in sweet words. It could not work. "[HCP, Female]*

> *"When we have time to inform about ART that we could not say more such as ART could suppress HIV and could not cure of HIV. Of course, we would like to inform patients and community completely. However, we should be wise to consider our targeted individuals. We say ART is a drug because if we send all information, patients and community could remember only the small part of the end our information." [HCP, Male]*

Provider factors

Willingness to help

In general, the willingness of HCPs to help patients was strongly articulated from both patients and HCPs. For example, HCPs took into account personal circumstances to help patients. One HCP said,

> *"If their home is far from the hospital, particularly for patients who fly by plane, we can give them 2 or 3 months of their ART. I appreciated a pregnant woman who travelled by bus from Sarmi [the name of a district] to here. I can see her commitment to prevent her baby from having HIV." [HCP, Female]*

On the other hand, HCPs had to consider the consequences of their help such as providing more supply of ART to one patient. One HCP said,

> *"Then after giving the stock for three months, we should be cautious about the stock for other patients. Giving three months ART for patients means we borrow from other patient's stock." [HCP, Female]*

Another HCP provided other help such as creating a safe environment in the clinic, transportation cost and providing food, as one explained.

> *"Here, I feel safe, everybody knows each other and I don't need to hassle and to collect*

For peer review only - http://bmjopen.bmj.com/site/about/guidelines.xhtml

ART, I come here every two months."[Patient, Male]

However, patients also acknowledged the fear of rejection and being stigmatized. One patient explained:

> *"How they treated me so bad, I must fight in emergency department after they rejected me. I was there not for free. I am Papuan, and I have rights to get health access. Finally, one doctor came then I said to him that I did not want others in that hospital to discriminate more. It was enough." [Patient, Female]*

Competence of HCPs and trust in HCP

Patients recognized the competence of HCPs, e.g., to provide information about medications. Patients described the benefits of ART. A patient described why she needed ART for the rest of her life.

> *'If someone is asking me about ART, I can let them know that ART can suppress my virus but not to cure. I need to take this tablet for a lifetime. My nurses explained this often to me." [Patient, Female]*

Similarly, HCPs perceived they had power to make patients feel inferior and dependent on health care system without any possibility to protest.

> *"I felt hurt, when I saw my colleagues treating patients while laughing and sometimes they were busy with their mobile during consultation."[HCP, Female]*

One patient revealed her situation when she wanted to receive information from her health providers

> *"I kept my mouth suddenly, when I saw her face. I could not open my mouth even I really needed to ask her about my problem." [Patient, Female]*

One healthcare provider revealed problems with confidential patient information. It was commonly found that HCPs refused to treat patients and asked other colleagues to replace their shift in preparing medication.

> "*A few weeks ago, we had problems with our staff. One of our staff was unable to maintain patient's status. He told the status of the patient to the family although the patient asked us to keep it confidential. He was not ready yet to disclose the status. I can understand if*

For peer review only

he disappeared and did not collect medication for a few months. But everything is now solved. " *[HCP, Female]*

DISCUSSION

In this qualitative study in Papua province, a region with a high prevalence of HIV and a relatively low uptake of ART, we explored Papuan's experiences of taking ART from the patient and the HCP perspective. We found that patient's knowledge, beliefs and routines and the health care environment had an impact on the medication taking of patients.

In line with a previous study we found that just the availability of ART is insufficient for patients to cope with their chronic HIV treatment [28]**.** In recent years, health care has been reorganized, so that HIV care can be provided in remote areas by *Puskesmas*, but some patients do not use those because of a lack of privacy. As in other studies, fear of disclosure of HIV status and the stigma associated with disclosure was an important barrier to accept this care closer to home [29]. This was not only found to be a barrier to get supply with ART, but also to discuss concerns and get information on the disease and treatment [28],[30].

Contrary to a previous study [31], patients valued the competence of HCPs highly. Nonetheless, both HCPs and patients reported communication barriers. An example was the difficulties to describe side effects of ART with sufficient detail, since the local language lacked the terms to do so. In addition, patients experienced the attitude of some HCPs as degrading. Examples included HCPs laughing or not paying attention to patients or discussing sensitive issues like genitals directly and loudly, as have been shown in other studies [8], [32]. Similar as in other studies, use of educational materials producing fear was also perceived to contribute to stigma [9],[33]. Addressing these issues in training of HCPs and developing educational materials suitable for the local population seems to be a first step to tackle these problems.

Patients and HCPs agreed that support from family, HCP and peers were important for the patients to cope with treatment. In particular the support of the family in medication taking was seen as essential as in other studies [34]–[36]. However despite support, patients could be feeling isolated by their family [37]. This condition may lead them to keep their HIV status as a secret. As in other studies, peer support was found to help patients [38]. But peers should receive education and training, because there is a risk of misinforming patients [39]. Furthermore, HCPs experienced difficulties to find sufficient patients who wanted to support

For peer review only - http://bmjopen.bmj.com/site/about/guidelines.xhtml

For peer review only

their peers because of fear to disclose their status. HCPs recognized that support should be personalized for each patient.

Religion was an important topic to be discussed for patients and HCPs. Contrary to a previous study [39], the religious beliefs empowered patients to take ART and being hopeful. Religious beliefs were also an important motivator for HCPs to offer the best service to patients. However, religious beliefs also contributed to the notion that HIV was a punishment due to personal failure.

This study highlighted that feeling stigmatized was widespread among PLHIV as stigma seemed to be the underlying problem for many of the issues that we identified [40]. A stigma reduction program seems to be needed to maximize the effects of existing HIV policies and provision of ART. There is very limited information how to successfully reduce HIV stigma in Indonesia. However, some interventions among people affected with leprosy in Indonesia [41], [42] might be good examples to be implemented. Testimony, and counselling, significantly reduced internal and external stigma among leprosy patients, and the community. Therefore, testimonies of HIV infected women took ART and were successful in having babies with no HIV infection might change the perceptions of community and healthcare providers. Furthermore, strengthening the economic situation of leprosy patients by providing microfinance has been shown to be successful. Similar with previous studies [43], [44], people who were in a better socioeconomic situation had better self-esteem and were actively involved with their community and had less internal stigma.

Strengths and Limitations

It should be noted that there were some limitations in this study. First, we specifically recruited patients from five different tribes in Papua. Other racial/ethnic groups and may have different experiences. Since we only included few participants from each tribe, we were not able to ascertain similarities and differences between tribes. Our data shows the overall experiences of PLHIV in this region. Second, we asked HCPs to ask patients to participate in this study, so our results refer to patients who remain in chronic care. More work needs to be done to identify factors driving patients to drop out of care completely. Third, recruiting patients through their health care professionals creates the risk that patients feel coerced to participate in the study. Because of the topic and non-obtrusive nature of this study, we believe this risk was minimal, but we cannot exclude this completely. Despite these limitations, our findings provide new

For peer review only - http://bmjopen.bmj.com/site/about/guidelines.xhtml

insights into the daily struggle in taking ART among Melanesians in Indonesia. Our findings are the basis for quantitative studies to identify how widespread some of the issues are. Furthermore, our findings should inspire others to explore the local context which is important to know to develop strategies to strengthen primary health care in many parts of the world [45].

CONCLUSION

Our findings are important for the further development of interventions to support patients in chronic treatment with ART taking into account the specific cultural needs of Papuan with HIV. In particular, addressing communication barriers is important. The HIV treatment strategies implemented in Papua need to take into account the local social and religious culture of Papuans. Broader strategies to reduce stigma should be a priority for the centralized and local government. This may remove some of the barriers for LHV to use the primary health centers. Hopefully, this can improve retention and adherence, and ultimately, health outcomes for Papuans living with HIV.

Competing interests

The authors have indicated that they have no competing interests

Authors' contributions

Conceived and designed the study: EIS, KT, EL, CE, AP. Analyzed the data: EIS, KT, EL, CE, AP. Wrote the paper: EIS, KT, EL, CE, AP. All authors read and approved the final manuscript

Acknowledgements

The authors would like to express their gratitude to Papuans living with HIV and healthcare providers who participated in this study.

Funding

This work was supported by Directorate General of Higher Education (DIKTI) Scholarship provided to EIS. The funding sponsor had no role in the design of study; in the collection, analyses, or interpretation of the results; in writing section, and the decision to publish this.

Ethics and Consent to participate

For peer review only - http://bmjopen.bmj.com/site/about/guidelines.xhtml

This study was approved from the Committee on the Ethics Commission, Faculty of Medicine, Public Health, and Nursing Universitas Gadjah Mada, Yogyakarta Indonesia (project number: KE/FK/0507/EC/2018).

Patient consent

All participants, patients and healthcare providers, gave written informed consent.

Data sharing statement

The data would not be shared outside of participating research institutions. Any queries on how to access the data set should be to the corresponding author or ira_sianturi@yahoo.co.id

Consent for publication

Not Applicable

References

[1] C. Murray, K. Ortblad, C. Guinovart, and E. Al, "Global, regional, and national incidence and mortality for HIV, tuberculosis, and malaria during 1990–2013: a systematic analysis for the Global Burden of Disease Study 2013," *Lancet*, vol. 20, no. 384, pp. 1005–1070, 2014.

[2] UN Joint Programme on HIV/AIDS (UNAIDS), "Global AIDS Update: Miles to go—closing gaps, breaking barriers, righting injustices.," *Report*, p. 268, 2018.

[3] B. L. Genberg, Y. Lee, W. H. Rogers, and I. B. Wilson, "Four types of barriers to adherence of antiretroviral therapy are associated with decreased adherence over time," *AIDS Behav.*, vol. 19, no. 1, pp. 85–92, 2015.

[4] S. G. Dalmida *et al.*, "Examination of the Role of Religious and Psychosocial Factors in HIV Medication Adherence Rates," *J. Relig. Health*, vol. 56, no. 6, pp. 2144–2161, 2017.

[5] Anonymous, "Indonesia Demographic and Health Survey 2012," 2013.

[6] E. N. Arifin, A. Ananta, D. R. W. W. Utami, N. B. Handayani, and A. Pramono, "Quantifying Indonesia's Ethnic Diversity: Statistics at National, Provincial, and District levels," *Asian Popul. Stud.*, vol. 11, no. 3, pp. 233–256, 2015.

[7] Dinas Kesehatan Provinsi Papua, "Profil Kesehatan Provinsi Papua Tahun 2016," 2017.

[8] A. Simonin, J. Bushee, and A. Courcaud, "Social, cultural and political factors in the

For peer review only - http://bmjopen.bmj.com/site/about/guidelines.xhtml

design of HIV programmes with Papuan highland communities," *Cult. Health Sex.*, vol. 13, no. sup2, pp. S185–S199, 2011.

[9] L. Butt, "'Lipstick Girls' and 'Fallen Women': AIDS and Conspiratorial Thinking in Papua, Indonesia," *Cult. Anthropol.*, vol. 20, no. 3, pp. 412–442, 2005.

[10] Y. Sugandi, "Conflict Analysis and Policy Recommendation on Papua," 2008.

[11] L. Butt, "Local Biologies and HIV/AIDS in Highlands Papua, Indonesia," *Cult. Med. Psychiatry*, vol. 37, no. 1, pp. 179–194, 2013.

[12] H. M. Kendrick, "Are religion and spirituality barriers or facilitators to treatment for HIV : a systematic review of the literature," *AIDS Care*, vol. 29, no. 1, pp. 1–13, 2017.

[13] K. Williams, B. G. Haire, S. Nathan, K. Williams, B. G. Haire, and S. Nathan, "' They say God punishes people with HIV ': experiences of stigma and discrimination among adults with HIV in Dili , Timor-Leste," *Cult. Health Sex.*, vol. 1058, pp. 1–14, 2017.

[14] L. Butt, J. Morin, G. Numbery, I. Eyon, and A. Goo, "Stigma and HIV / AIDS in Highlands Papua," 2010.

[15] L. Butt, G. Numbery, and J. Morin, "The smokescreen of culture: AIDS and the indigenous in Papua, Indonesia.," *Pac. Health Dialog*, vol. 9, no. 2, pp. 283–9, 2002.

[16] R. Granich, S. Gupta, J. Montaner, B. Williams, and J. M. Zuniga, "Pattern, Determinants, and Impact of HIV Spending on Care and Treatment in 38 High-Burden Low- and Middle-Income Countries," *J. Int. Assoc. Provid. AIDS Care*, vol. 15, no. 2, pp. 91–100, 2016.

[17] F. M. Ekawati, M. Claramita, K. Hort, J. Furler, S. Licqurish, and J. Gunn, "Patients' experience of using primary care services in the context of Indonesian universal health coverage reforms," *Asia Pac. Fam. Med.*, vol. 16, no. 1, pp. 1–10, 2017.

[18] Kementerian Kesehatan Republik Indonesia - Direktorat Jenderal Pengendalian Penyakit dan Penyehatan Lingkungan, *Pedoman Nasional Tatalaksana Klinis Infeksi HIV dan Terapi Antiretroviral pada Orang Dewasa*. 2011.

[19] M. Desai, J. W. Rudge, W. Adisasmito, S. Mounier-Jack, and R. Coker, "Critical interactions between Global Fund-supported programmes and health systems: a case study in Indonesia.," *Health Policy Plan.*, 2010.

[20] Shreeshant Prabhakaran, Arin Dutta, Rebecca Ross, and Catherine Barker Cantelmo, "Options To Finance The Rapid Scale-Up Of The HIV Response In Indonesia The Role of the National Health Insurance Scheme (JKN) and Local Governments," 2018.

[21] Anonymous, "Laporan Perkembangan HIV AIDS dan PIMS Triwulan 3 2018.pdf," 2018.

For peer review only - http://bmjopen.bmj.com/site/about/guidelines.xhtml

[22] Anonymous (Kementerian Kesehatan Republik Indonesia), *Petunjuk Teknis: Pengisian Formulir Kesehatan Pencatatan dan Pelaporan Program Pengendalian HIV AIDS dan IMS*. 2015.

[23] Kementerian Kesehatan Republik Indonesia, "Laporan Perkembangan HIV-AIDS & PIMS di Indonesia Triwulan 1 tahun 2019," 2019.

[24] S. Rueda *et al.*, "Examining the associations between HIV-related stigma and health outcomes in people living with HIV/AIDS: a series of meta-analyses.," *BMJ Open*, vol. 6, no. 7, p. e011453, 2016.

[25] C. W. Holtzman *et al.*, "Mapping Patient–Identified Barriers and Facilitators to Retention in HIV Care and Antiretroviral Therapy Adherence to Andersen's Behavioral Model," *AIDS Care*, vol. 27, no. 7, pp. 817–828, 2015.

[26] L. Atuyambe, S. Neema, E. Otolok-Tanga, G. Wamuyu-Maina, S. Kasasa, and F. Wabwire-Mangen, "The effects of enhanced access to antiretroviral therapy: A qualitative study of community perceptions in Kampala city, Uganda," *Afr. Health Sci.*, vol. 8, no. 1, pp. 13–19, 2008.

[27] R. M. Anderson, "Revisiting the Behavioral Model and Access to Medical Care : Does it Matter ?," *J. Health Soc. Behav.*, vol. 36, no. 1, pp. 1–10, 1995.

[28] S. Treves-Kagan *et al.*, "Why increasing availability of ART is not enough: A rapid, community-based study on how HIV-related stigma impacts engagement to care in rural South Africa," *BMC Public Health*, vol. 16, no. 1, pp. 1–13, 2016.

[29] F. S. Shabalala *et al.*, "Understanding reasons for discontinued antiretroviral treatment among clients in test and treat: a qualitative study in Swaziland," *J. Int. AIDS Soc.*, vol. 21, p. e25120, 2018.

[30] M. Sugiana, I. N. Sutarsa, and D. P. Duarsa, "Barriers to Integrating Antiretroviral Therapy Services Into Community Health Centre : A Qualitative Study in Badung Regency Hambatan-hambatan Puskesmas Sebagai Satelit Antiretroviral Therapy (ART) : Penelitian Kualitatif di Kabupaten Badung," *Public Heal. Prev. Med. Arch.*, vol. 3, no. 1, pp. 60–65, 2015.

[31] T. O. Famoroti, L. Fernandes, and S. C. Chima, "Stigmatization of people living with HIV/AIDS by healthcare workers at a tertiary hospital in KwaZulu-Natal, South Africa: A cross-sectional descriptive study," *BMC Med. Ethics*, vol. 14, no. SUPPL.1, pp. 1–10, 2013.

[32] F. van Loggerenberg *et al.*, "A Qualitative Study of Patient Motivation to Adhere to Combination Antiretroviral Therapy in South Africa," *AIDS Patient Care STDS*, vol.

For peer review only - http://bmjopen.bmj.com/site/about/guidelines.xhtml

29, no. 5, pp. 299–306, 2015.

[33] M. Flassy, “Deficit of Women ’ s Human Right in Papua Province : A Conceptual Framework,” vol. 2, no. 2, pp. 28–52, 2019.

[34] S. P. Wasti *et al.*, “Factors influencing adherence to antiretroviral treatment in Asian developing countries: A systematic review,” *Trop. Med. Int. Heal.*, vol. 17, no. 1, pp. 71–81, 2012.

[35] J. Kibicho and J. Owczarzak, “A Patient-Centered Pharmacy Services Model of HIV Patient Care in Community Pharmacy Settings: A Theoretical and Empirical Framework,” *AIDS Patient Care STDS*, vol. 26, no. 1, pp. 20–28, 2011.

[36] J. F. Xu, Z. Q. Ming, Y. Q. Zhang, P. C. Wang, J. Jing, and F. Cheng, “Family support, discrimination, and quality of life among ART-treated HIV-infected patients: A two-year study in China,” *Infect. Dis. Poverty*, vol. 6, no. 1, pp. 1–10, 2017.

[37] S. Park and K. S. Park, “Family stigma: A concept analysis,” *Asian Nurs. Res. (Korean. Soc. Nurs. Sci).*, vol. 8, no. 3, pp. 165–171, 2014.

[38] R. Karwa *et al.*, “Leveraging peer-based support to facilitate HIV care in Kenya,” *PLOS Med.*, vol. 14, no. 7, p. e1002355, 2017.

[39] S. Kulkarni *et al.*, “Identifying Perceived Barriers along the HIV Care Continuum: Findings from Providers, Peer Educators, and Observations of Provider–Patient Interactions in Ethiopia,” *J Int. Assoc Provid AIDS Care*, vol. 15, no. 4, pp. 291–300, 2016.

[40] H. A. Cross, M. Heijnders, A. Dalal, S. Sermrittirong, and S. Mak, “Interventions for stigma reduction-part 2: Practical applications,” *Asia Pacific Disabil. Rehabil. J.*, vol. 22, no. 3, pp. 71–80, 2011.

[41] R. M. H. Peters, Dadun, M. B. M. Zweekhorst, J. F. G. Bunders, Irwanto, and W. H. van Brakel, “A Cluster-Randomized Controlled Intervention Study to Assess the Effect of a Contact Intervention in Reducing Leprosy-Related Stigma in Indonesia,” *PLoS Negl. Trop. Dis.*, vol. 9, no. 10, pp. 1–24, 2015.

[42] M. Lusli *et al.*, “The Impact of a Rights-Based Counselling Intervention to Reduce Stigma in People Affected by Leprosy in Indonesia,” *PLoS Negl. Trop. Dis.*, vol. 10, no. 12, pp. 1–25, 2016.

[43] D. Dadun *et al.*, “Impact of socio-economic development, contact and peer counselling on stigma against persons affected by leprosy in Cirebon, Indonesia - a randomised controlled trial,” *Lepr. Rev.*, vol. 88, no. 1, pp. 2–22, 2017.

[44] D. Dadun, R. M. H. Peters, W. H. van Brakel, J. G. F. Bunders, I. Irwanto, and B. J.

For peer review only - http://bmjopen.bmj.com/site/about/guidelines.xhtml

Regeer, "Assessing the impact of the twin track socio-economic intervention on reducing leprosy-related stigma in Cirebon District, Indonesia," *Int. J. Environ. Res. Public Health*, vol. 16, no. 3, pp. 1–20, 2019.

[45] UNICEF, "A vision for primary health care in the 21st century," 2018.

For peer review only

For peer review only - http://bmjopen.bmj.com/site/about/guidelines.xhtml

Table 1. Characteristics of study participants (n=27)

Participant	Characteristics	Number (%)	*Mamta (Jayapura)*	*Mee Pago (Timika)*	*Saereri (Serui)*	*Anin Ha (Merauke)*	*LaPago (Wamena)*
Healthcare providers (n=14)	Mean age 42 (range:35-55 years)						
	Work at Hospital	8 (57%)	1	1	3	2	1
	Work at Community health center (*Puskesmas*)	6 (43%)	2	2	1	0	1
	Female	9 (64%)	1	3	2	1	2
	Male	5 (36%)	2	0	2	1	0
	Physician	3 (12%)	1	0	1	1	0
	Midwife	1 (7%)	0	0	1	0	0
	Nurse	9 (64%)	2	3	1	1	2
	Psychologist	1 (7%)	0	0	1	0	0
Patients (n=13)	Mean age 33 (range:20-60 years)						
	Medication dispensed from hospital pharmacy	10 (77%)	3	0	3	3	1
	Medication dispensed from community health center (*Puskesmas*)	3 (23%)	0	2	0	0	1
	Female	8 (61%)	2	1	2	2	1
	Male	5 (39%)	1	1	1	1	1
	Diagnosis following VCT	11 (85%)	3	2	2	3	1
	Diagnosis on antenatal ward	2 (15%)	0	0	1	0	1

VCT: Voluntary, Counselling, and Testing; *Puskesmas*: *Pusat Kesehatan Masyarakat*

For peer review only - http://bmjopen.bmj.com/site/about/guidelines.xhtml

Appendix Interview Guide

Main questions:

1. What were the health problems forcing you to test your HIV status?

2. What were your experience before and after HCPs placed you on ART?

Probing Question (not necessarily being asked, depends on answer from the main question):

1. *How long you have been initiated ART since you were diagnosed with HIV?*
2. *How convenient is it to get ART in this facility?*
3. *Have you planned to access another facility?*
4. *In your view, do you think HIV/AIDS is still a big threat in this community?*
5. *Where do most HIV/AIDS patients seek care?*
6. *What type of care is available for HIV/AIDS patients in this community?*
7. *What sort of treatment is usually given to HIV/AIDS patients in this area?*
8. *What are some of the beliefs regarding risk to HIV infection with increased availability of ART?*
9. *What prevention measures against HIV/AIDS do people in this area use (probe for condom use, reducing sexual partners, abstinence, etc.)*
10. *What is the community's attitude towards HIV prevention with availability of ART for AIDS treatment?*

For peer review only

Consolidated criteria for reporting qualitative studies (COREQ): 32-item checklist

Manuscript:

The daily struggle to take antiretrovirals: a qualitative study in Papuans living with HIV and their healthcare providers

No. Item	Guide questions/description	Reported on Page #	Checklists
**Domain 1: Research team and reflexivity**			
Personal Characteristics			
1. Inter viewer/facilitator	Which author/s conducted the inter view or focus group?	Methods	√
2. Credentials	What were the researcher's credentials? E.g. PhD, MD	Methods	√
3. Occupation	What was their occupation at the time of the study?	Methods	√
4. Gender	Was the researcher male or female?	Methods	√
5. Experience and training	What experience or training did the researcher have?	Methods	√
Relationship with participants			
6. Relationship established	Was a relationship established prior to study commencement?	N/A	
7. Participant knowledge of the interviewer	What did the participants know about the researcher? e.g. personal goals, reasons for doing the research	Methods	√
8. Interviewer characteristics	What characteristics were reported about the inter viewer/facilitator? e.g. Bias, assumptions, reasons and interests in the research topic	Methods	√
**Domain 2: study design**			
Theoretical framework			
9. Methodological orientation and Theory	What methodological orientation was stated to underpin the study? e.g. grounded theory, discourse analysis, ethnography, phenomenology, content analysis	Methods	√
Participant selection			
10. Sampling	How were participants selected? e.g. purposive, convenience, consecutive, snowball	Methods	√

For peer review only

11. Method of approach	How were participants approached? e.g. face-to-face, telephone, mail, email	Methods	√
12. Sample size	How many participants were in the study?	Results	√
13. Non-participation	How many people refused to participate or dropped out? Reasons?	Results	√
Setting			
14. Setting of data collection	Where was the data collected? e.g. home, clinic, workplace	Methods	√
15. Presence of non-participants	Was anyone else present besides the participants and researchers?	Methods	√
16. Description of sample	What are the important characteristics of the sample? e.g. demographic data, date	Methods and Results	√
Data collection			
17. Interview guide	Were questions, prompts, guides provided by the authors? Was it pilot tested?	Methods	√
18. Repeat interviews	Were repeat inter views carried out? If yes, how many?	N/A	
19. Audio/visual recording	Did the research use audio or visual recording to collect the data?	Methods	√
20. Field notes	Were field notes made during and/or after the inter view or focus group?	Methods	√
21. Duration	What was the duration of the inter views or focus group?	Methods	√
22. Data saturation	Was data saturation discussed?	Methods	√
23. Transcripts returned	Were transcripts returned to participants for comment and/or correction?	Methods	√
**Domain 3: analysis and findings**			
Data analysis			
24. Number of data coders	How many data coders coded the data?	Methods	√
25. Description of the coding tree	Did authors provide a description of the coding tree?	Appendix	√
26. Derivation of themes	Were themes identified in advance or derived from the data?	Methods and appendix	√
27. Software	What software, if applicable, was used to manage the data?	Atlas Ti	√
28. Participant checking	Did participants provide feedback on the findings?	N/A	
Reporting			
29. Quotations presented	Were participant quotations presented to illustrate the themes/findings? Was each quotation identified? e.g.	Results	√

For peer review only

	participant number		
30. Data and findings consistent	Was there consistency between the data presented and the findings?	N/A	
31. Clarity of major themes	Were major themes clearly presented in the findings?	Results	√
32. Clarity of minor themes	Is there a description of diverse cases or discussion of minor themes?	Discussion	√

For peer review only

BMJ Open

The daily struggle to take antiretrovirals: a qualitative study in Papuans living with HIV and their healthcare providers

Journal:	*BMJ Open*
Manuscript ID	bmjopen-2020-036832.R2
Article Type:	Original research
Date Submitted by the Author:	27-Jul-2020
Complete List of Authors:	Sianturi, Elfride; Groningen University Faculty of Mathematics and Natural Sciences, Groningen Research Institute of Pharmacy Unit PharmacoTherapy, -Epidemiology & -Economics (PTEE); Universitas Cenderawasih,<br>Latifah, E; Universitas Muhammadiyah Magelang<br>Probandari, Ari; Universitas Sebelas Maret, Department of Public Health, Faculty of Medicine<br>Effendy, C; Gadjah Mada University Faculty of Medicine<br>Taxis, Katja; Groningen University Faculty of Mathematics and Natural Sciences, Groningen Research Institute of Pharmacy Unit PharmacoTherapy, -Epidemiology & -Economics (PTEE)
**Primary Subject Heading**:	Qualitative research
Secondary Subject Heading:	Health policy, Public health, Sociology
Keywords:	HIV & AIDS < INFECTIOUS DISEASES, Quality in health care < HEALTH SERVICES ADMINISTRATION & MANAGEMENT, EPIDEMIOLOGY, Health policy < HEALTH SERVICES ADMINISTRATION & MANAGEMENT, PRIMARY CARE

SCHOLARONE™
Manuscripts

I, the Submitting Author has the right to grant and does grant on behalf of all authors of the Work (as defined in the below author licence), an exclusive licence and/or a non-exclusive licence for contributions from authors who are: i) UK Crown employees; ii) where BMJ has agreed a CC-BY licence shall apply, and/or iii) in accordance with the terms applicable for US Federal Government officers or employees acting as part of their official duties; on a worldwide, perpetual, irrevocable, royalty-free basis to BMJ Publishing Group Ltd ("BMJ") its licensees and where the relevant Journal is co-owned by BMJ to the co-owners of the Journal, to publish the Work in this journal and any other BMJ products and to exploit all rights, as set out in our licence.

The Submitting Author accepts and understands that any supply made under these terms is made by BMJ to the Submitting Author unless you are acting as an employee on behalf of your employer or a postgraduate student of an affiliated institution which is paying any applicable article publishing charge ("APC") for Open Access articles. Where the Submitting Author wishes to make the Work available on an Open Access basis (and intends to pay the relevant APC), the terms of reuse of such Open Access shall be governed by a Creative Commons licence – details of these licences and which Creative Commons licence will apply to this Work are set out in our licence referred to above.

Other than as permitted in any relevant BMJ Author's Self Archiving Policies, I confirm this Work has not been accepted for publication elsewhere, is not being considered for publication elsewhere and does not duplicate material already published. I confirm all authors consent to publication of this Work and authorise the granting of this licence.

For peer review only

The daily struggle to take antiretrovirals: a qualitative study in Papuans living with HIV and their healthcare providers

E.I Sianturi[1,2,] E. Latifah[3], A. Probandari[4], C. Effendy[5], K. Taxis[1]

[1]PharmacoTherapy,-Epidemiology&-Economics (PTEE) Department of Pharmacy, University of Groningen, the Netherlands

[2]Faculty of Mathematics and Natural Sciences University of Cenderawasih, Papua-Indonesia

[3]Department of Pharmacy, Faculty of Health Science, Universitas Muhammadiyah Magelang, Magelang, Indonesia

[4]Department of Public Health, Faculty of Medicine, Universitas Sebelas Maret, Jl. Ir. Sutami 36A, Surakarta, 57126, Indonesia.

[5]School of Nursing, Faculty of Medicine, Public Health and Nursing, Universitas Gadjah Mada, Yogyakarta, Indonesia.

Correspondence should be addressed to:

E.I Sianturi, Antonius Deusinglaan 1, 9713 AV, Groningen, The Netherlands, Contact Phone Number: +31 0622820392, Fax number: +31 503637576; Contact email: e.i.sianturi@rug.nl; ira_sianturi@yahoo.co.id

Email's authors:

E.I Sianturi: ira_sianturi@yahoo.co.id; e.i.sianturi@rug.nl

E. Latifah: elmiawatilatifah@ummgl.ac.id

A. Probandari: ariprobandari@yahoo.com

C. Effendy: christantie@ugm.ac.id

K. Taxis: k.taxis@rug.nl

For peer review only

ABSTRACT

Objective: The aim of the study was to explore the experiences of Papuans living with HIV to take Antiretroviral Therapy (ART) from the patient and the health care providers (HCPs) perspective.

Design: This was a qualitative study covering all five tribes located in Papua Provinces one of two Indonesian provinces on Papua Island. Semi structured interviews were conducted with Papuans living with HIV and their HCPs. Interviews were transcribed verbatim and coded to find themes.

Results: Overall, we conducted interviews with 13 Papuans living with HIV (mean age: 33 years, 61 % female) and 14 HCPs (mean age: 42 years, 64% female) within five customary areas. HCPs included three physicians, nine nurses, two others. Two main themes were identified: (1) personal factors, and (2) healthcare environment-related factors. Personal factors were beliefs and knowledge of ART, stigma from family, community, and HCPs as well as practical problems such as transportation because of long distance. Within the theme of healthcare environment, the competences and attitudes of HCPs were particularly relevant. The findings are important in refining HIV treatment strategies implemented in Papua, especially when extending HIV care provided by community centers.

Conclusions: Despite free access to ART, Papuans living with HIV struggle to remain on treatment. Considering local culture and religion in strategies to reduce stigma should be a priority.

Keyword: HIV, Papuan, Melanesian, tribes, customary-area, local culture, stigma, Indonesia

For peer review only

Strengths and Limitations of this study

- The findings provide new insights into the daily struggle in taking ART among Melanesians in Indonesia.
- This study is the basis for quantitative studies to identify how widespread some of the issues are and the findings should inspire others to explore the local context which is important to know to develop strategies to strengthen primary health care in many parts of the world.
- This study specifically recruited patients from five different tribes in Papua. Other racial/ethnic groups may have different experiences.
- Recruiting patients through their health care professionals may create the risk that patients feel coerced to participate in the study.

For peer review only

BACKGROUND

Antiretroviral therapy (ART) has saved millions of people living with HIV (PLHIV) [1]. Treatment is lifelong. Despite such successes, access to treatment and retention in care is still an issue in many parts of the world [2]. Economic barriers, stigma, social relationships, religion, local concepts about illness and medication impact on the care for PLHIV [3], [4].

Indonesia consists of more than 17 thousand islands and has been viewed as one of Southeast Asia's highest performing economies in recent decades [5]. A large number of islands has fostered the development of a diverse culture with more than four hundred ethnic groups, each with their own language(s) [5], [6]. The prevalence of HIV is about 0.1% across Indonesia. The prevalence in Papua is about 2.3%, with the highest prevalence found in the remote areas of the highlands [7]. Papua Island consists of two provinces, Papua and West Papua. Ethnically, most Papuans are Melanesians belonging to one of six distinct local tribes, with Christianity being the dominant religion. This is in contrast to the rest of Indonesia where Islam is the dominant religion. Within Indonesia, Papua has a special autonomy status due to high revenues from the exploitation of natural resources [2], [8], [9]. In many instances, Papuans are viewed as being primitive in a globalized world [8]. The feeling of being stigmatized seems to be common among Papuans and it has been growing due to inequalities and poor security levels [10]. In particular, feeling stigmatized has been found in PLHIV since being infected means breaking cultural norms [11]–[13]. A prior study showed that stigma among female Papuans with HIV was even more pronounced than male Papuans [14].

Despite the status of a special autonomous region, challenges remain to organize health care for Papuans living with HIV because of the large geographical area, the lack of infrastructure and transportation into many parts, especially the remote highlands. Other challenges include the high number of illiterates, especially in the remote areas [15] and the local concepts of illness, death, and misfortune which include the widely held belief that death and sickness occur intentionally [8], [15]. Such cultural concepts may result in communication barriers between health care workers who are mainly migrants from other areas of Indonesia and the native Papuans [16]. Health care for PLHIV was initially provided by hospitals, but in recent years, is also provided by community health centers [16], [17].

ART is provided free of charge in Papua [18]. To ensure the sustainability of the ART supply chain [19]–[21], every PLHIV must be registered in the national cohort before initiation of ART

For peer review only - http://bmjopen.bmj.com/site/about/guidelines.xhtml

[22]. Even though there were 153 locations which provided HIV care in Papua Province [23], less than 25% of Papuans living with HIV were on ART [7], [23]. The coverage of ART in Papua was lower than in other parts of Indonesia [2]. Previous survey-based studies in Papua showed low levels of health literacy [24], and lower levels of adherence to medication than in other parts of Indonesia [25]. We also found that enacted stigma was significantly associated with non-adherence among Papuans [26]. To improve the situation for Papuans living with HIV, more in-depth knowledge is needed on barriers and facilitators to access medication and remain on treatment in particular in the changing organization of health care taking into account the local culture. The aim of the study was to explore the experiences of Papuans living with HIV to take ART from the patient and the HCP perspective.

METHODS

Study design, setting and population

This was an exploratory qualitative study. Data were collected between July and September 2018. The study covered all five customary areas in Papua Province, namely LaPago (Wamena), Mee Pago (Timika), both from the highland areas and Anin Ha (Merauke), Saereri (Serui), and Mamta (Jayapura) from the lowland areas. We included patients who received ART either from a hospital or from a community health center, and HCPs working in HIV care. Each site included at least two patients; one male and one female, and two HCPs. The inclusion criteria of patients were: (1) native Papuans or migrants having a Papuan spouse, (2) aged ≥18 years, (3) were on antiretroviral therapy for at least one year before commencing the interview, and (4) willing and able to give informed consent. The inclusion criteria of HCPs were: (1) working in HIV care for at least two years in a hospital or in a community health center (called *Puskesmas* in Indonesia), (2) having completed training in HIV care, and (3) willing and able to give informed consent. The study was approved by the Ethics Commission, Faculty of Medicine, Public Health, and Nursing Universitas Gadjah Mada (number: KE/FK/0507/EC/2018). Before the interviews, participants were informed about the study purposes and ethical approval and signed consent was obtained from each participant. We assured all participants that data would be kept confidential and participants would have no disadvantages in receiving health care. This paper was written following the Consolidated Criteria for Reporting Qualitative Research (COREQ) guideline for reporting [27].

For peer review only - http://bmjopen.bmj.com/site/about/guidelines.xhtml

Study procedure, data collection, and management of data

The interview guide was developed based on literature [28], [29]. The research team of this study had a diverse background from pharmacy (EIS, EL, KT), epidemiologist (AP), and nursing field (CE) and all researchers are female. Two with a masters degree and three with a PhD degree. The interviews were conducted by EIS and two research assistants with a background in sociology and nursing who have worked for almost 25 years in HIV care in Jayapura. Interviews were conducted in Bahasa Indonesia which is the national language of Indonesia. All interviews took place in a private location as agreed between participants and interviewers, and lasted approximately one hour. Topics covered in the interviews were the health problems which triggered getting a test of their HIV status, and the patient's experiences before and after starting to take ART.

We audio-recorded the interviews. The interviewers also took field notes during the interviews. All audio recordings were transcribed verbatim, leaving out noise sounds and conversation outside of the study topics. We limited transcription to 3 interviews per day per transcriber to ensure thoroughness. All transcripts were double-checked. We offered the participants to read the transcripts, but none of the participations wished to do so. Each transcript was given a unique identifier composed of the type of participant, and gender.

Data analysis

Data were analyzed using the Atlas Ti Version 8.4 software. Transcripts were coded by EIS, EL, CE taking a similar approach as in a previous study. We chose a content-oriented approach to analyse the data (undirected coding). All transcripts were read and re-read to develop an initial coding framework which was refined during coding. Once the coding was completed, categories were developed. Themes were identified from the categories. Disagreements in the analysis were resolved by discussions between all authors. The themes were finally mapped with an existing larger framework [28], [30]. We stopped interviewing when we reached saturation within 2 participant groups.

Patient and public involvement

EIS has worked as a HCP in Papua previously and therefore knew HPCs in all facilities. EIS contacted potentially eligible HCPs in all facilities and invited them to participate in the study. HCPs agreed to participate were also asked to contact potentially eligible patients to participate in this study. The participants gave written informed consent before interviews.

For peer review only - http://bmjopen.bmj.com/site/about/guidelines.xhtml

RESULTS

Participants

All HCPs and PLHIV who were approached agreed to participate in the study. Overall, 14 HCPs participated who recruited 13 patients (Table 1). The 14 HCPs were three physicians, nine nurses, one psychologist, and one midwife. The mean age of HCPs was 42 years. Among HCPs, 9 were female and 8 worked in hospital. Ten out of 13 Papuan patients got their medication from the hospital pharmacy, and the remaining patients accessed *Puskesmas* to collect their ART. The mean age of patients was 33 years. Among the patients, 8 were females, and 11 patients were diagnosed in a Voluntary, Counseling, and Testing (VCT) clinic following ongoing symptoms of illness. There was no participant who was contacted refused to participate this study. Additional participant's characteristic shows in Table 1.

The codes and categories were mapped on the two themes from existing frameworks [28],[30]. The themes were: (1) factors that represented patient's personal experiences of taking ART and (2) the descriptions of the healthcare environment which played a role in taking ART.

Theme 1: Patient Factors

We identified personal beliefs and knowledge about ART, religion, verbal and nonverbal communication, finances and transport, social support, and other responsibilities as being important factors for patients in dealing with taking ART.

Personal beliefs and knowledge about ART

A patient who previously dropped out of care described that they believed in traditional medicine being helpful. The patient also believed ART was a not a drug since ART did not cure the symptoms and the disease.

> *"My doctor said that my ART required a lifetime commitment. Then I thought ART was just to extend my life not for making my virus disappear…"[Patient, Male]*

An HCP explained insufficient knowledge has challenged patients to try alternative medication.

> *"The insufficient knowledge is a problem for the patients. I found patients attempting to use traditional medication since they had the lack of knowledge of ART. I think it was because we informed them that their ART is to suppress their virus not to eliminate. "[HCP, Female]*

For peer review only - http://bmjopen.bmj.com/site/about/guidelines.xhtml

Differently, the patient's knowledge level played a role in coping with HIV.

Religion

Most participants took religion seriously. Christian belief shaped attitudes of participants to accept HIV as their disease. Another patient took hope from religious belief that HIV could be cured. They believed that taking ART was the way for God to help them.

> *" God's miracle will happen, just believe and take ART regularly." [Patient, Male]*

In a similar way, HCPs used religion to measure the quality of their service and believed helping patients was the same as serving God.

> *"I told my colleagues, our salary and incentives would be bloody money, when we were not honest in doing our job. Our money would be like water into our noken (woven bag). It disappears fast because we did not work according to God's will. We are blessed if we treat our patients likely what God wants." [HCP, Female]*

In contrast, both patients and HCPs also shared their views that based on the religion patients were immoral, and God used HIV as a warning sign to sinners.

> *"I think there is a link between HIV and religion. Patients were infected with the virus because they were not afraid to of God's law. They were not married yet, drunk and had sex with women who were not their partner." [HCP, Female]*

Verbal and nonverbal communication

Participants stressed it was important to talk about genital terms in prudent ways as this is important for Papuans. Besides verbal language, patients reported about non-verbal signs such as unprofessional behavior of personnel and breaches of confidentiality. One patient strongly articulated his experiences with a HCP. Laughing and not being seriously while informing about disease related to sexual transmitted disease was found and irritated PLHIV.

> *"A nurse increased her voice when giving us information…it might be her character…. However, I preferred that others who did not have business with my health could not hear" [Patient, Male]*

The language HCPs used was mentioned as an issue. For example, HCPs had problems to express medical terms about side effects in simple words, especially HCPs who were not native Papuans.

For peer review only - http://bmjopen.bmj.com/site/about/guidelines.xhtml

"I think language is challenging here. I tried counselling them about side effects, such as rash, or stiffness but I could not find words to replace those into their language. So, sometimes I must say "kaskado" to replace rash." [HCP, Female]

The gap in language has also produced misunderstandings in the community, especially in the HIV awareness program. One patient shared his experience about this program before he was diagnosed with HIV as follows.

"The nurses came here often to give some programs about the HIV awareness. They said many things, we listened but we got nothing. We did not understand …."[Patient, Male]

Finance and transport

Patients weighed the costs and benefits of being treated in *Puskesmas* however some patients preferred the hospital despite having to spend more money and time**.**

"I needed about IDR [Indonesian rupiah] 30.000-60.000 every time to collect my ART at a hospital. It may be costlier than collecting ART at Puskesmas, which only take 10.000 IDR, but I decided to keep collecting ART here in hospital." [Patient, Female]

In general, participants said that distance and transportation cost were barriers to keep taking ART.

"I had been waiting for the trucks which would bring me to the city to collect my ART. Nevertheless, my boss and the trucks never came, and I heard that there was a problem with gasoline stock supply." [Patient, Male]

Social support from family and peers

We found that patients and HCPs had different opinions about social support. In general, a positive relationship with others was described as powerful to support patients over time and reduce the risk of discontinuation of ART. There was both, acceptance and rejection from families. Even though it happened, HCPs considered having family support was essential to be present before they placed patients on ART. The HCPs believed the existence of family would help patients to overcome fear about medication and social problems.

"Most of the patients who had a good relationship with family, they are adherent and there is only a small percentage of them who stopped their ART. However, for patients who hide their status, most of them found their lives as useless and they could stop ART

For peer review only - http://bmjopen.bmj.com/site/about/guidelines.xhtml

For peer review only

anytime. The hindrances do not really impact patients when they know their family protect them." [HCP, Female]

Patients revealed different feelings and experiences about the family's support. They expressed that the family influenced the decision to seek health care. There could be a delay in being diagnosed and be on ART because of the family. One patient mentioned her family brought her to hospital, which was far away from her house. It was impossible to bring her to the nearest health facility because her father also worked there.

In order to receive help from their family, patients needed to disclose their status. To disclose HIV status was described as a double-edged sword, either to receive support or being stigmatized.

"My family know my HIV, they support me to take my medicine, but they have separated my plate, glasses from others."[Patient, Male]

Participants also reported about their experiences with support from peers, i.e. other PLHIV. In some locations formal or informal peer-support groups exist. Some patients described that knowing peers helped them to cope with fear and empower them. With peers, patients were not alone anymore and sometimes peers linked them with better HIV care.

"I join peers. I am happy with that. I can meet people who have the same condition with me. We discussed many things, not only medication but how we deal with stress. With them I can discuss many things including something private, ha ha…about our genitals." [Patient, Female]

One patient revealed that a HCP asked her to visit patients who did not attend the hospital for collecting ART.

"In my situation, HCP asked me to visit other patients who did not attend the hospital. HCP considered my visit to patient's house could not raise suspicions. All my expenses to visit other patients were paid by HCP." [Patient, Female]

HCPs said that the influence of peers may be positive or negative.

"Sometimes, I was disappointed with some peers. They persuaded my patients who were adherent to move to other health facilities and asked them to try herbals. I know it was not 100% peer's faults, my patients could not filter obtained information but the reason

For peer review only - http://bmjopen.bmj.com/site/about/guidelines.xhtml

we introduced peers to patients was to help others but to not make others their followers." [HCP, Female]

Other responsibilities

A number of difficulties keeping to the regular medication intake schedule were identified. These included being bored or having other responsibilities, e.g., work-related activities which made it difficult to take ART. One patient said,

"I don't think so that my ART is not important, but I must sell 'pinang' (betel nut) every day to support my life and my daughter. I worked until late at night and it made me felt tired and sleepy. Then I forgot to take my medication. So if I have a break I take my medication directly." [Patient, Female]

Theme 2: Health Care Environment

Within the theme of health care environment, we identified health service and health care providers as important factors which influenced the experiences of patients in taking ART. Infrastructure and perceived complicated administrative system were most commonly reported as health service-related factors. Participants also shared their experiences with the home visit programme and educational services. Provider factors consisted of willingness to help, competence of HCPs and trust in HCP.

Health service factors

Infrastructure

Both patients and HCPs reported *Puskesmas* and hospital had the facilities to diagnose HIV. Some patients did not know that it was possible to collect ART in *Puskesmas*. Even though there is the possibility of collecting ART in *Puskesmas*, this option was rejected by some patients. Patients found the infrastructure and laboratory monitoring insufficient. A community center with only a small room used for all patients irrespective of their disease created fear of being exposed as HIV positive to others.

"I think the performance of personnel in Puskemas is good. However, the consultation room is small and without separation. Everyone can hear what nurses are saying… I think everyone can HCPs *recognize my HIV directly." [Patient, Male]*

For peer review only - http://bmjopen.bmj.com/site/about/guidelines.xhtml

The under-utilization of *Puskesmas* has been considered as problems for patients and HCPs. The HCPs were frustrated because they realized that their goal of improving the quality of health services could not be achieved.

"Since I have worked in Puskesmas, I have found the lack of laboratory is still a problem. We want that Puskesmas can be independent and our goal to deliver the high quality of health service can be achieved. I do not know when it is going to happen." [HCP, Female]

Perceived complicated administration system

Patients and HCPs mentioned administration as an important topic. Most patients could not understand why HCPs asked them to show their single identity number (NIK= *Nomor Induk Kependudukan*). The patients thought that this was irrelevant since costs for health care should be covered. One patient expressed her worries about this:

"M*y concern was only about my card…… My nurses have asked it many times and I could not show it." [Patient, Female]*

Some HCPs explained that patients needed to register with their NIK to be included in the national HIV cohort to receive ART. Without NIK, a delay in starting ART could occur.

Home visit programme and educational services

Patients and HCPs also discussed the effectiveness of the home visit programs. HCP's perspective revealed that they needed to attach some pictures as proof to get reimbursement from their health insurance. However, patients felt uncomfortable about the HCPs taking pictures in their homes. Also, patients felt the home visits increased the suspicion in the community. Some patients offered to have meetings with HCPs in another place but not in their house.

"I remembered one of my patients refused my visit to his house. He said to me….' please do not come…. I am afraid people will be curious about your visit'. He offered to meet me in other place instead of his house." [HCP, Female]

Sometimes HCPs found their colleagues documented their activities to make fictitious visits. One HCP shared,

"Most of our colleagues tried making fictitious programs. They made some photos as complementary documents to get reimbursement. Most of our colleagues focused on money and they had many tricks to get reimbursement." [HCP, Female]

For peer review only - http://bmjopen.bmj.com/site/about/guidelines.xhtml

Some HCPs noted that they often had a deterrent effect when promoting the HIV program in community because they used scary images of skeletons. The concept of HIV could be treated with medication was omitted from the program.

> *"In early days, we used images of death, skeleton and disfigurement in our HIV awareness program. We hope the community can get a deterrent effect. Later on, the community can learn about how to prevent it. However, we found the information about the HIV syndrome was among the topics most commonly remembered by community. I think most people are stubborn. It is better to use a deterrent effect. We could not say our information about HIV in sweet words. It could not work. "[HCP, Female]*

> *"When we have time to inform about ART that we could not say more such as ART could suppress HIV and could not cure of HIV. Of course, we would like to inform patients and community completely. However, we should be wise to consider our targeted individuals. We say ART is a drug because if we send all information, patients and community could remember only the small part of the end our information." [HCP, Male]*

Provider factors

Willingness to help

In general, the willingness of HCPs to help patients was strongly articulated from both patients and HCPs. For example, HCPs took into account personal circumstances to help patients. One HCP said,

> *"If their home is far from the hospital, particularly for patients who fly by plane, we can give them 2 or 3 months of their ART. I appreciated a pregnant woman who travelled by bus from Sarmi [the name of a district] to here. I can see her commitment to prevent her baby from having HIV." [HCP, Female]*

On the other hand, HCPs had to consider the consequences of their help such as providing more supply of ART to one patient. One HCP said,

> *"Then after giving the stock for three months, we should be cautious about the stock for other patients. Giving three months ART for patients means we borrow from other patient's stock." [HCP, Female]*

Another HCP provided other help such as creating a safe environment in the clinic, transportation cost and providing food, as one explained.

For peer review only - http://bmjopen.bmj.com/site/about/guidelines.xhtml

"Here, I feel safe, everybody knows each other and I don't need to hassle and to collect ART, I come here every two months."[Patient, Male]

However, patients also acknowledged the fear of rejection and being stigmatized. One patient explained:

"How they treated me so bad, I must fight in emergency department after they rejected me. I was there not for free. I am Papuan, and I have rights to get health access. Finally, one doctor came then I said to him that I did not want others in that hospital to discriminate more. It was enough." [Patient, Female]

Competence of HCPs and trust in HCP

Patients recognized the competence of HCPs, e.g., to provide information about medications. Patients described the benefits of ART. A patient described why she needed ART for the rest of her life.

'If someone is asking me about ART, I can let them know that ART can suppress my virus but not to cure. I need to take this tablet for a lifetime. My nurses explained this often to me." [Patient, Female]

Similarly, HCPs perceived they had power to make patients feel inferior and dependent on health care system without any possibility to protest.

"I felt hurt, when I saw my colleagues treating patients while laughing and sometimes they were busy with their mobile during consultation."[HCP, Female]

One patient revealed her situation when she wanted to receive information from her health providers.

"I kept my mouth suddenly, when I saw her face. I could not open my mouth even I really needed to ask her about my problem." [Patient, Female]

One healthcare provider revealed problems with confidential patient information. It was commonly found that HCPs refused to treat patients and asked other colleagues to replace their shift in preparing medication.

"*A few weeks ago, we had problems with our staff. One of our staff was unable to maintain patient's status. He told the status of the patient to the family although the patient asked us to keep it confidential. He was not ready yet to disclose the status. I can understand if*

For peer review only - http://bmjopen.bmj.com/site/about/guidelines.xhtml

he disappeared and did not collect medication for a few months. But everything is now solved. " [HCP, Female]

DISCUSSION

In this qualitative study in Papua province, a region with a high prevalence of HIV and a relatively low uptake of ART, we explored Papuan's experiences of taking ART from the patient and the HCP perspective. We found that patient's knowledge, beliefs and routines and the health care environment had an impact on the medication taking of patients.

In line with a previous study we found that just the availability of ART is insufficient for patients to cope with their chronic HIV treatment [31]**.** In recent years, health care has been reorganized, so that HIV care can be provided in remote areas by *Puskesmas*, but some patients do not use those because of a lack of privacy. As in other studies, fear of disclosure of HIV status and the stigma associated with disclosure was an important barrier to accept this care closer to home [32]. This was not only found to be a barrier to get supply with ART, but also to discuss concerns and get information on the disease and treatment [31],[33].

Contrary to a previous study [34], patients valued the competence of HCPs highly. Nonetheless, both HCPs and patients reported communication barriers. An example was the difficulties to describe side effects of ART with sufficient detail, since the local language lacked the terms to do so. In addition, patients experienced the attitude of some HCPs as degrading. Examples included HCPs laughing or not paying attention to patients or discussing sensitive issues like genitals directly and loudly, as have been shown in other studies [8], [35]. Similar as in other studies, use of educational materials producing fear was also perceived to contribute to stigma [9],[36]. Since having training before HCPs placed is mandatory, this study recommends stigma reduction should be available in one of training topics. Addressing these issues in training of HCPs and developing educational materials suitable for the local population seems to be a first step to tackle these problems.

Patients and HCPs agreed that support from family, HCP and peers were important for the patients to cope with treatment. In particular the support of the family in medication taking was seen as essential as in other studies [37]–[39]. However despite support, patients could be feeling isolated by their family [40]. This condition may lead them to keep their HIV status as a secret. As in other studies, peer support was found to help patients [41]. But peers should receive education and training, because there is a risk of misinforming patients [42].

For peer review only - http://bmjopen.bmj.com/site/about/guidelines.xhtml

Furthermore, HCPs experienced difficulties to find sufficient patients who wanted to support their peers because of fear to disclose their status. HCPs recognized that support should be personalized for each patient.

Religion was an important topic to be discussed for patients and HCPs. Contrary to a previous study [42], the religious beliefs empowered patients to take ART and being hopeful. Religious beliefs were also an important motivator for HCPs to offer the best service to patients. However, religious beliefs also contributed to the notion that HIV was a punishment due to personal failure.

This study highlighted that feeling stigmatized was widespread among PLHIV as stigma seemed to be the underlying problem for many of the issues that we identified [43]. A stigma reduction program seems to be needed to maximize the effects of existing HIV policies and provision of ART. There is very limited information how to successfully reduce HIV stigma in Indonesia. However, some interventions among people affected with leprosy in Indonesia [44], [45] might be good examples to be implemented. Testimony, and counselling, significantly reduced internal and external stigma among leprosy patients, and the community. Therefore, testimonies of HIV infected women took ART and were successful in having babies with no HIV infection might change the perceptions of community and healthcare providers. Furthermore, strengthening the economic situation of leprosy patients by providing microfinance has been shown to be successful. Similar with previous studies [46], [47], people who were in a better socioeconomic situation had better self-esteem and were actively involved with their community and had less internal stigma.

Strengths and Limitations

It should be noted that there were some limitations in this study. First, we specifically recruited patients from five different tribes in Papua. Other racial/ethnic groups and may have different experiences. Since we only included few participants from each tribe, we were not able to ascertain similarities and differences between tribes. Our data shows the overall experiences of PLHIV in this region. Second, we asked HCPs to ask patients to participate in this study, so our results refer to patients who remain in chronic care. More work needs to be done to identify factors driving patients to drop out of care completely. Third, recruiting patients through their health care professionals creates the risk that patients feel coerced to participate in the study. Because of the topic and non-obtrusive nature of this study, we believe this risk was minimal,

For peer review only - http://bmjopen.bmj.com/site/about/guidelines.xhtml

but we cannot exclude this completely. Despite these limitations, our findings provide new insights into the daily struggle in taking ART among Melanesians in Indonesia. Our findings are the basis for quantitative studies to identify how widespread some of the issues are. Furthermore, our findings should inspire others to explore the local context which is important to know to develop strategies to strengthen primary health care in many parts of the world [48].

CONCLUSION

Our findings are important for the further development of interventions to support patients in chronic treatment with ART taking into account the specific cultural needs of Papuan with HIV. In particular, addressing communication barriers is important. The HIV treatment strategies implemented in Papua need to take into account the local social and religious culture of Papuans. Broader strategies to reduce stigma should be a priority for the centralized and local government. This may remove some of the barriers for LHV to use the primary health centers. Hopefully, this can improve retention and adherence, and ultimately, health outcomes for Papuans living with HIV.

Competing interests

The authors have indicated that they have no competing interests

Authors' contributions

Conceived and designed the study: EIS, KT, EL, CE, AP. Analyzed the data: EIS, KT, EL, CE, AP. Wrote the paper: EIS, KT, EL, CE, AP. All authors read and approved the final manuscript

Acknowledgements

The authors would like to express their gratitude to Papuans living with HIV and healthcare providers who participated in this study.

Funding

This work was supported by Directorate General of Higher Education (DIKTI) Scholarship provided to EIS (No:15.6/E.4.4/2015). The funding sponsor had no role in the design of study; in the collection, analyses, or interpretation of the results; in writing section, and the decision to publish this.

Ethics and Consent to participate

For peer review only - http://bmjopen.bmj.com/site/about/guidelines.xhtml

This study was approved from the Committee on the Ethics Commission, Faculty of Medicine, Public Health, and Nursing Universitas Gadjah Mada, Yogyakarta Indonesia (project number: KE/FK/0507/EC/2018).

Patient consent

All participants, patients and healthcare providers, gave written informed consent.

Data sharing statement

The data would not be shared outside of participating research institutions. Any queries on how to access the data set should be to the corresponding author or ira_sianturi@yahoo.co.id

Consent for publication

Not Applicable

References

[1] C. Murray, K. Ortblad, C. Guinovart, and E. Al, "Global, regional, and national incidence and mortality for HIV, tuberculosis, and malaria during 1990–2013: a systematic analysis for the Global Burden of Disease Study 2013," *Lancet*, vol. 20, no. 384, pp. 1005–1070, 2014.

[2] UN Joint Programme on HIV/AIDS (UNAIDS), "Global AIDS Update: Miles to go—closing gaps, breaking barriers, righting injustices.," *Report*, p. 268, 2018.

[3] B. L. Genberg, Y. Lee, W. H. Rogers, and I. B. Wilson, "Four types of barriers to adherence of antiretroviral therapy are associated with decreased adherence over time," *AIDS Behav.*, vol. 19, no. 1, pp. 85–92, 2015.

[4] S. G. Dalmida *et al.*, "Examination of the Role of Religious and Psychosocial Factors in HIV Medication Adherence Rates," *J. Relig. Health*, vol. 56, no. 6, pp. 2144–2161, 2017.

[5] Anonymous, "Indonesia Demographic and Health Survey 2012," 2013.

[6] E. N. Arifin, A. Ananta, D. R. W. W. Utami, N. B. Handayani, and A. Pramono, "Quantifying Indonesia's Ethnic Diversity: Statistics at National, Provincial, and District levels," *Asian Popul. Stud.*, vol. 11, no. 3, pp. 233–256, 2015.

[7] Dinas Kesehatan Provinsi Papua, "Profil Kesehatan Provinsi Papua Tahun 2016," 2017.

[8] A. Simonin, J. Bushee, and A. Courcaud, "Social, cultural and political factors in the

For peer review only - http://bmjopen.bmj.com/site/about/guidelines.xhtml

design of HIV programmes with Papuan highland communities," *Cult. Health Sex.*, vol. 13, no. sup2, pp. S185–S199, 2011.

[9] L. Butt, "'Lipstick Girls' and 'Fallen Women': AIDS and Conspiratorial Thinking in Papua, Indonesia," *Cult. Anthropol.*, vol. 20, no. 3, pp. 412–442, 2005.

[10] Y. Sugandi, "Conflict Analysis and Policy Recommendation on Papua," 2008.

[11] L. Butt, "Local Biologies and HIV/AIDS in Highlands Papua, Indonesia," *Cult. Med. Psychiatry*, vol. 37, no. 1, pp. 179–194, 2013.

[12] H. M. Kendrick, "Are religion and spirituality barriers or facilitators to treatment for HIV : a systematic review of the literature," *AIDS Care*, vol. 29, no. 1, pp. 1–13, 2017.

[13] K. Williams, B. G. Haire, S. Nathan, K. Williams, B. G. Haire, and S. Nathan, "' They say God punishes people with HIV ': experiences of stigma and discrimination among adults with HIV in Dili , Timor-Leste," *Cult. Health Sex.*, vol. 1058, pp. 1–14, 2017.

[14] L. Butt, J. Morin, G. Numbery, I. Eyon, and A. Goo, "Stigma and HIV / AIDS in Highlands Papua," 2010.

[15] L. Butt, G. Numbery, and J. Morin, "The smokescreen of culture: AIDS and the indigenous in Papua, Indonesia.," *Pac. Health Dialog*, vol. 9, no. 2, pp. 283–9, 2002.

[16] R. Granich, S. Gupta, J. Montaner, B. Williams, and J. M. Zuniga, "Pattern, Determinants, and Impact of HIV Spending on Care and Treatment in 38 High-Burden Low- and Middle-Income Countries," *J. Int. Assoc. Provid. AIDS Care*, vol. 15, no. 2, pp. 91–100, 2016.

[17] F. M. Ekawati, M. Claramita, K. Hort, J. Furler, S. Licqurish, and J. Gunn, "Patients' experience of using primary care services in the context of Indonesian universal health coverage reforms," *Asia Pac. Fam. Med.*, vol. 16, no. 1, pp. 1–10, 2017.

[18] Kementerian Kesehatan Republik Indonesia - Direktorat Jenderal Pengendalian Penyakit dan Penyehatan Lingkungan, *Pedoman Nasional Tatalaksana Klinis Infeksi HIV dan Terapi Antiretroviral pada Orang Dewasa*. 2011.

[19] M. Desai, J. W. Rudge, W. Adisasmito, S. Mounier-Jack, and R. Coker, "Critical interactions between Global Fund-supported programmes and health systems: a case study in Indonesia.," *Health Policy Plan.*, 2010.

[20] Shreeshant Prabhakaran, Arin Dutta, Rebecca Ross, and Catherine Barker Cantelmo, "Options To Finance The Rapid Scale-Up Of The HIV Response In Indonesia The Role of the National Health Insurance Scheme (JKN) and Local Governments," 2018.

[21] Anonymous, "Laporan Perkembangan HIV AIDS dan PIMS Triwulan 3 2018.pdf," 2018.

For peer review only - http://bmjopen.bmj.com/site/about/guidelines.xhtml

[22] Anonymous (Kementerian Kesehatan Republik Indonesia), *Petunjuk Teknis: Pengisian Formulir Kesehatan Pencatatan dan Pelaporan Program Pengendalian HIV AIDS dan IMS*. 2015.

[23] Kementerian Kesehatan Republik Indonesia, "Laporan Perkembangan HIV-AIDS & PIMS di Indonesia Triwulan 1 tahun 2019," 2019.

[24] E. I. Sianturi, D. A. Perwitasari, S. N. Soltief, M. Atiqul Islam, B. Geboers, and K. Taxis, "Health literacy of people living with HIV in a rural area in Indonesia: A cross-sectional study," *Heal. Soc. Care Community*, no. May, pp. 1–8, 2020.

[25] E. I. Sianturi, D. A. Perwitasari, A. Islam, and K. Taxis, "The association between ethnicity , stigma , beliefs about medicines and adherence in people living with HIV in a rural area in Indonesia," *BMC Public Health*, pp. 1–8, 2019.

[26] S. Rueda *et al.*, "Examining the associations between HIV-related stigma and health outcomes in people living with HIV/AIDS: a series of meta-analyses.," *BMJ Open*, vol. 6, no. 7, p. e011453, 2016.

[27] A. Tong, P. Sainsbury, and J. Craig, "Consolidated criteria for reporting qualitative research (COREQ): A 32-item checklist for interviews and focus groups," *Int. J. Qual. Heal. Care*, vol. 19, no. 6, pp. 349–357, 2007.

[28] C. W. Holtzman *et al.*, "Mapping Patient–Identified Barriers and Facilitators to Retention in HIV Care and Antiretroviral Therapy Adherence to Andersen's Behavioral Model," *AIDS Care*, vol. 27, no. 7, pp. 817–828, 2015.

[29] L. Atuyambe, S. Neema, E. Otolok-Tanga, G. Wamuyu-Maina, S. Kasasa, and F. Wabwire-Mangen, "The effects of enhanced access to antiretroviral therapy: A qualitative study of community perceptions in Kampala city, Uganda," *Afr. Health Sci.*, vol. 8, no. 1, pp. 13–19, 2008.

[30] R. M. Anderson, "Revisiting the Behavioral Model and Access to Medical Care : Does it Matter ?," *J. Health Soc. Behav.*, vol. 36, no. 1, pp. 1–10, 1995.

[31] S. Treves-Kagan *et al.*, "Why increasing availability of ART is not enough: A rapid, community-based study on how HIV-related stigma impacts engagement to care in rural South Africa," *BMC Public Health*, vol. 16, no. 1, pp. 1–13, 2016.

[32] F. S. Shabalala *et al.*, "Understanding reasons for discontinued antiretroviral treatment among clients in test and treat: a qualitative study in Swaziland," *J. Int. AIDS Soc.*, vol. 21, p. e25120, 2018.

[33] M. Sugiana, I. N. Sutarsa, and D. P. Duarsa, "Barriers to Integrating Antiretroviral Therapy Services Into Community Health Centre : A Qualitative Study in Badung

For peer review only - http://bmjopen.bmj.com/site/about/guidelines.xhtml

Regency Hambatan-hambatan Puskesmas Sebagai Satelit Antiretroviral Therapy (ART) : Penelitian Kualitatif di Kabupaten Badung," *Public Heal. Prev. Med. Arch.*, vol. 3, no. 1, pp. 60–65, 2015.

[34] T. O. Famoroti, L. Fernandes, and S. C. Chima, "Stigmatization of people living with HIV/AIDS by healthcare workers at a tertiary hospital in KwaZulu-Natal, South Africa: A cross-sectional descriptive study," *BMC Med. Ethics*, vol. 14, no. SUPPL.1, pp. 1–10, 2013.

[35] F. van Loggerenberg *et al.*, "A Qualitative Study of Patient Motivation to Adhere to Combination Antiretroviral Therapy in South Africa," *AIDS Patient Care STDS*, vol. 29, no. 5, pp. 299–306, 2015.

[36] M. Flassy, "Deficit of Women ' s Human Right in Papua Province : A Conceptual Framework," vol. 2, no. 2, pp. 28–52, 2019.

[37] S. P. Wasti *et al.*, "Factors influencing adherence to antiretroviral treatment in Asian developing countries: A systematic review," *Trop. Med. Int. Heal.*, vol. 17, no. 1, pp. 71–81, 2012.

[38] J. Kibicho and J. Owczarzak, "A Patient-Centered Pharmacy Services Model of HIV Patient Care in Community Pharmacy Settings: A Theoretical and Empirical Framework," *AIDS Patient Care STDS*, vol. 26, no. 1, pp. 20–28, 2011.

[39] J. F. Xu, Z. Q. Ming, Y. Q. Zhang, P. C. Wang, J. Jing, and F. Cheng, "Family support, discrimination, and quality of life among ART-treated HIV-infected patients: A two-year study in China," *Infect. Dis. Poverty*, vol. 6, no. 1, pp. 1–10, 2017.

[40] S. Park and K. S. Park, "Family stigma: A concept analysis," *Asian Nurs. Res. (Korean. Soc. Nurs. Sci).*, vol. 8, no. 3, pp. 165–171, 2014.

[41] R. Karwa *et al.*, "Leveraging peer-based support to facilitate HIV care in Kenya," *PLOS Med.*, vol. 14, no. 7, p. e1002355, 2017.

[42] S. Kulkarni *et al.*, "Identifying Perceived Barriers along the HIV Care Continuum: Findings from Providers, Peer Educators, and Observations of Provider–Patient Interactions in Ethiopia," *J Int. Assoc Provid AIDS Care*, vol. 15, no. 4, pp. 291–300, 2016.

[43] H. A. Cross, M. Heijnders, A. Dalal, S. Sermrittirong, and S. Mak, "Interventions for stigma reduction-part 2: Practical applications," *Asia Pacific Disabil. Rehabil. J.*, vol. 22, no. 3, pp. 71–80, 2011.

[44] R. M. H. Peters, Dadun, M. B. M. Zweekhorst, J. F. G. Bunders, Irwanto, and W. H. van Brakel, "A Cluster-Randomized Controlled Intervention Study to Assess the Effect

For peer review only - http://bmjopen.bmj.com/site/about/guidelines.xhtml

of a Contact Intervention in Reducing Leprosy-Related Stigma in Indonesia," *PLoS Negl. Trop. Dis.*, vol. 9, no. 10, pp. 1–24, 2015.

[45] M. Lusli *et al.*, "The Impact of a Rights-Based Counselling Intervention to Reduce Stigma in People Affected by Leprosy in Indonesia," *PLoS Negl. Trop. Dis.*, vol. 10, no. 12, pp. 1–25, 2016.

[46] D. Dadun *et al.*, "Impact of socio-economic development, contact and peer counselling on stigma against persons affected by leprosy in Cirebon, Indonesia - a randomised controlled trial," *Lepr. Rev.*, vol. 88, no. 1, pp. 2–22, 2017.

[47] D. Dadun, R. M. H. Peters, W. H. van Brakel, J. G. F. Bunders, I. Irwanto, and B. J. Regeer, "Assessing the impact of the twin track socio-economic intervention on reducing leprosy-related stigma in Cirebon District, Indonesia," *Int. J. Environ. Res. Public Health*, vol. 16, no. 3, pp. 1–20, 2019.

[48] UNICEF, "A vision for primary health care in the 21st century," 2018.

For peer review only

Table 1. Characteristics of study participants (n=27)

Participant	Characteristics	Number (%)	*Mamta (Jayapura)*	*Mee Pago (Timika)*	*Saereri (Serui)*	*Anin Ha (Merauke)*	*LaPago (Wamena)*
Healthcare providers (n=14)	Mean age 42 (range:35-55 years)						
	Work at Hospital	8 (57%)	1	1	3	2	1
	Work at Community health center (*Puskesmas*)	6 (43%)	2	2	1	0	1
	Female	9 (64%)	1	3	2	1	2
	Male	5 (36%)	2	0	2	1	0
	Physician	3 (12%)	1	0	1	1	0
	Midwife	1 (7%)	0	0	1	0	0
	Nurse	9 (64%)	2	3	1	1	2
	Psychologist	1 (7%)	0	0	1	0	0
Patients (n=13)	Mean age 33 (range:20-60 years)						
	Medication dispensed from hospital pharmacy	10 (77%)	3	0	3	3	1
	Medication dispensed from community health center (*Puskesmas*)	3 (23%)	0	2	0	0	1
	Female	8 (61%)	2	1	2	2	1
	Male	5 (39%)	1	1	1	1	1
	Diagnosis following VCT	11 (85%)	3	2	2	3	1
	Diagnosis on antenatal ward	2 (15%)	0	0	1	0	1

VCT: Voluntary, Counselling, and Testing; *Puskesmas*: *Pusat Kesehatan Masyarakat*

For peer review only

Consolidated criteria for reporting qualitative studies (COREQ): 32-item checklist

Manuscript:

The daily struggle to take antiretrovirals: a qualitative study in Papuans living with HIV and their healthcare providers

No. Item	Guide questions/description	Reported on Page #	Checklists
**Domain 1: Research team and reflexivity**			
Personal Characteristics			
1. Inter viewer/facilitator	Which author/s conducted the inter view or focus group?	Methods (6)	√
2. Credentials	What were the researcher's credentials? E.g. PhD, MD	Methods (6)	√
3. Occupation	What was their occupation at the time of the study?	Methods (6)	√
4. Gender	Was the researcher male or female?	Methods (6)	√
5. Experience and training	What experience or training did the researcher have?	Methods (6)	√
Relationship with participants			
6. Relationship established	Was a relationship established prior to study commencement?	N/A	
7. Participant knowledge of the interviewer	What did the participants know about the researcher? e.g. personal goals, reasons for doing the research	Methods (5)	√
8. Interviewer characteristics	What characteristics were reported about the inter viewer/facilitator? e.g. Bias, assumptions, reasons and interests in the research topic	Methods (6)	√
**Domain 2: study design**			
Theoretical framework			
9. Methodological orientation and Theory	What methodological orientation was stated to underpin the study? e.g. grounded theory, discourse analysis, ethnography, phenomenology, content analysis	Methods (5)	√
Participant selection			
10. Sampling	How were participants selected? e.g. purposive, convenience, consecutive, snowball	Methods (6)	√

For peer review only

11. Method of approach	How were participants approached? e.g. face-to-face, telephone, mail, email	Methods (6)	√
12. Sample size	How many participants were in the study?	Results (7)	√
13. Non-participation	How many people refused to participate or dropped out? Reasons?	Results (7)	√
Setting			
14. Setting of data collection	Where was the data collected? e.g. home, clinic, workplace	Methods (6)	√
15. Presence of non-participants	Was anyone else present besides the participants and researchers?	Methods (6)	√
16. Description of sample	What are the important characteristics of the sample? e.g. demographic data, date	Methods (6)	√
Data collection			
17. Interview guide	Were questions, prompts, guides provided by the authors? Was it pilot tested?	Methods (5)	√
18. Repeat interviews	Were repeat inter views carried out? If yes, how many?	N/A	
19. Audio/visual recording	Did the research use audio or visual recording to collect the data?	Methods (6)	√
20. Field notes	Were field notes made during and/or after the inter view or focus group?	Methods (6)	√
21. Duration	What was the duration of the inter views or focus group?	Methods (6)	√
22. Data saturation	Was data saturation discussed?	Methods (6)	√
23. Transcripts returned	Were transcripts returned to participants for comment and/or correction?	Methods (6)	√
**Domain 3: analysis and findings**			
Data analysis			
24. Number of data coders	How many data coders coded the data?	Methods (6)	√
25. Description of the coding tree	Did authors provide a description of the coding tree?	Methods (6)	
26. Derivation of themes	Were themes identified in advance or derived from the data?	Methods (6)	√
27. Software	What software, if applicable, was used to manage the data?	Methods (6)	√
28. Participant checking	Did participants provide feedback on the findings?	N/A	
Reporting			
29. Quotations presented	Were participant quotations presented to illustrate the themes/findings? Was each quotation identified? e.g. participant number	Results (7-15)	√

For peer review only

30. Data and findings consistent	Was there consistency between the data presented and the findings?	N/A	
31. Clarity of major themes	Were major themes clearly presented in the findings?	Results (7)	√
32. Clarity of minor themes	Is there a description of diverse cases or discussion of minor themes?	Discussion	√

For peer review only
